# Accelerated reprogramming of hiPSCs into functional brain endothelial-like cells using multiplexed CRISPR activation

**DOI:** 10.1038/s41598-026-46961-5

**Published:** 2026-04-09

**Authors:** Roy W. Hwang, Yara Khalil, Ewa Baumann, Junzhuo Huang, Claudie Charlebois, Marina Rukhlova, Tyler M. Renner, Ziying Liu, Anna Jezierski, Mads Kærn, Will J. Costain

**Affiliations:** 1 Faculty of Medicine, Department of Cellular Molecular Medicine Ottawa, University of Ottawa, Ottawa, ON K1H 8M5 Canada; 2 Faculty of Medicine, Department of Biochemistry, Microbiology and Immunology, University of Ottawa, Ottawa, ON K1H 8M5 Canada; 3 Human Health Therapeutics Research Centre, National Research Council of Canada, Ottawa, ON K1A 0R6 Canada; 4 Digital Technologies Research Centre, National Research Council of Canada, Ottawa, ON K1A 0R6 Canada; 5 Ottawa Institute of Systems Biology, University of Ottawa, Ottawa, ON K1H 8M5 Canada

**Keywords:** CRISPR, Brain endothelial cells, Blood-brain barrier, iPSC, Transcriptional reprogramming, Synthetic biology, Biotechnology, Cell biology, Developmental biology, Neuroscience, Stem cells

## Abstract

Human induced pluripotent stem cells (hiPSCs) offer a renewable and scalable source for generating brain endothelial cells (BECs), enabling the development of in vitro blood-brain barrier (BBB) models that closely reflect human physiology. In this study, we demonstrate a streamlined differentiation strategy for producing hiPSC-derived BEC-like cells (iBECs) using multiplex CRISPR/dCas9 activation (CRISPRa) to transcriptionally reprogram iPSCs by selectively and simultaneously upregulating BEC transcription factors (ERG, ETV2, FLI1) and BEC genes (CLDN5, CDH5, PECAM1, KDR). We observe that this approach significantly accelerates differentiation from 13 to five days while maintaining BBB characteristics. CRISPRa iBECs exhibit high transendothelial electrical resistance (TEER ~ 400 Ω·cm^2^), low transcellular permeability (< 0.027 × 10⁻^3^ cm/min), and expression of key BBB markers including Claudin-5, ZO-1, CDH5, PECAM1, Occludin, and GLUT-1, as well as receptor-mediated transporters TfR1, IGF1R, and TMEM30A. Our study demonstrates a novel CRISPRa-directed differentiation strategy that not only accelerates BEC differentiation but also demonstrates the utility of CRISPR gene regulation as an option in modifying the phenotype of iPSC-derived cells.

**Supplementary Information:**

The online version contains supplementary material available at 10.1038/s41598-026-46961-5.

## Introduction

In vitro blood-brain barrier (BBB) models are essential tools for investigating neurological diseases, central nervous system (CNS)-targeted drug delivery, and neuroinvasive pathogens^1–3^. The BBB is composed of brain endothelial cells (BECs), specialized cells that protect the central nervous system from potentially harmful blood-borne substances while regulating the selective transport of essential nutrients^4^. BBB dysfunction has been reported in many neurological conditions such as, Alzheimer’s disease, Huntington’s disease, and stroke^5^. Therefore, the development of physiologically accurate in vitro models that enable translatable mechanistic studies of CNS pathology and therapeutic intervention is of significant interest; however, BBB modeling is notoriously challenging due to the complexity of the neurovascular unit^6,7^. Primary BECs suffer from accessibility and reproducibility issues as their use involves receiving donor tissues from post-mortem patients from various backgrounds, health conditions and age^8^. To mitigate this, immortalized BECs have been developed^9,10^; however, immortalization of primary BECs can result in the loss of important BBB phenotypes and the expression of key marker proteins^9^. Alternatively, many researchers have turned to animal models to investigate the BBB as a structure and its implicit role in neurological diseases^11,12^. However, interspecies differences in protein sequences, transporter expression, and gene expression profiles can lessen the clinical translatability of non-human BBB models, particularly for antibody therapeutics^13–15^.

The advent of human induced-pluripotent stem cell (hiPSC) technology has enabled the production of human iPSC-derived BEC-like cells (iBECs)^16^. Differentiation protocols have been developed and optimized to produce phenotypes that are consistent with in vivo BEC properties and function, including high transendothelial electrical resistance (TEER), expression of brain endothelial-specific tight junction, adherens junction and adhesion markers, such as CLDN5, ZO-1, Occludin, GLUT1, CDH5 (VE-Cadherin) and PECAM1 (CD31), and functional polarized BBB transporters^17–20^. The translational relevance of iBEC models is demonstrated by isogenic systems that uncover disease-specific differences in BBB-associated markers and efflux transporter activity between iBECs derived from Alzheimer’s disease patients and healthy controls^21^. Although promising, recent studies have highlighted that many iBEC differentiation protocols result in incomplete maturation and oftentimes heterogeneous populations of endothelial and epithelial cell types, expressing epithelial markers such as EPCAM and E-Cadherin (epi-iBECs)^22^. Consequently, iBECs may not faithfully recapitulate the functional phenotypes intrinsic to primary BECs, such as expression of endothelial-specific transcriptional signatures, upregulation of immune adhesion molecules in response to inflammatory cues, and the ability to undergo angiogenesis.

Here, we demonstrate a novel differentiation scheme that involves transcriptionally reprogramming hiPSCs with essential endothelial marker genes and ETS transcription factors^23^ using a multiplex CRISPR activation (CRISPRa) approach. Our CRISPRa implementation simultaneously activates multiple genes by multiplexing optimized single guide RNAs (sgRNAs) to induce and accelerate BEC differentiation with a defined BEC phenotype. Our strategy demonstrates that hiPSCs can efficiently be directly reprogrammed into a BEC fate in under five days using established differentiation protocols^24^, exhibiting functional TEER, low paracellular and transcellular permeability, tight junction expression, angiogenesis and the expression of receptor-mediated transcytosis proteins. This study demonstrates the potential of CRISPR-mediated transcriptional reprogramming as an alternative strategy for stem cell differentiation platforms.

## Results

### CRISPRa screening of guide RNAs for brain endothelial gene targets

We identified seven genes involved in brain endothelial differentiation and phenotype that are typically under-expressed in iPSC-derived BEC models: three transcription factors (ERG, ETV2, FLI1) and four genes important to BEC function (CLDN5, CDH5, PECAM1, KDR) were selected for CRISPR-mediated activation in iPSCs^25^. The four selected BEC genes function in barrier formation, membrane transport and angiogenesis, all inherent phenotypes essential to modeling the BBB. Furthermore, the panel of transcription factors are known to play an essential role in angiogenesis and BEC differentiation^25^ (Table 1).

**Table 1:** Genetic markers for brain endothelial cell lineage reprogramming. Data derived from Lu_2021^22^.

Genetic markers	Gene group
ERG	ETS transcription factor
ETV2	ETS transcription factor
FLI1	ETS transcription factor
CLDN5	BEC marker
PECAM1	BEC marker
CDH5	BEC marker
KDR	BEC marker
*SOX7, SOX17, SOX18	Transcription factor

*Genes reported in subsequent studies and not included in original construct.

**Fig. 1:**
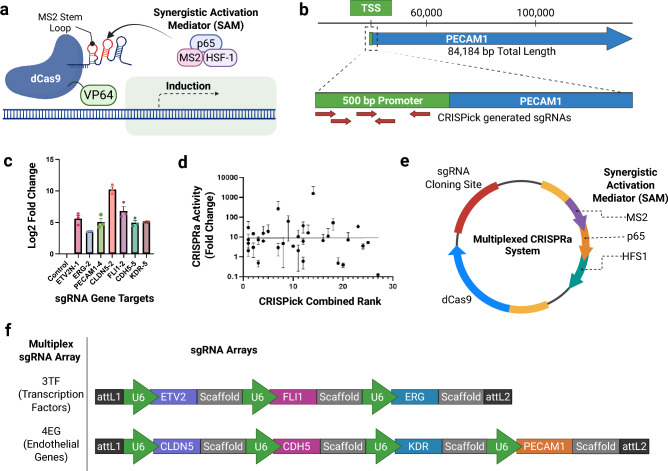
Identifying and screening brain endothelial gene targets and sgRNAs for CRISPRa in HeLa Cells. (**a**) Graphical illustration of CRISPRa utilizing the synergistic activation mediator (SAM) system. (**b**) Schematic overview of how various sgRNAs targeting the same gene were designed with Broad Institute’s CRISPick software. Candidate sgRNAs were specifically selected within 500 bp upstream of the transcription start site of each target gene. (**c**) Screening for putative highly performing sgRNA designs for each key brain endothelial gene target in HeLa cells co-transfected with CRISPRa and sgRNA vectors. RT-qPCR was performed for each HeLa transfected culture to identify which sgRNA elicited a consistently robust response. Individual Ct values were assessed in biological triplicates (n = 3) and normalized to Log_2_ fold change (mean ± SEM). (**d**) Correlation plot of observed CRISPRa performance (y-axis) and expected performance based on of the combined rank predicted by CRISPick algorithm (x-axis) (mean ± SEM). (**e**) Schematic of all-in-one multiplexed CRISPRa vector. (**f**) Tandem sgRNA designs targeting core BBB genes and transcription factors. Final engineered sgRNA designs are 3TF and 4EG.

Among the described CRISPRa systems, we selected the synergistic activation mediator (SAM) system based on its ability to be multiplexed and its described consistency in transcriptional activation performance in mammalian cell lines^26^ (Fig. 1a). For each gene, we used CRISPick (Broad Institute) to design five sgRNAs that target the genomic region immediately upstream of the respective transcription start sites (Fig. 1b) (Table S1 and S5). The performance (induction of gene expression) of individual sgRNAs was initially assessed in HeLa cells by determining relative mRNA expression by RT-qPCR (Figure S1). Optimal individual sgRNAs for each gene were selected for inclusion in the multiplexed CRISPRa construct (Fig. 1c). Interestingly, we observed a high degree of variability in gene activation among individual sgRNAs targeting a given gene in HeLa cells, as well as a lack of correlation between gene induction and the CRISPick sgRNA ranking algorithm (Fig. 1d). Thus, identification of optimal sgRNAs based on observed efficacy in CRISPRa/i systems appears to be an important step to ensure functionality.

### Constructing a multiplexed CRISPRa system targeting BEC genes

We used the one-step piggyBac transposase (PB)-based multiplex system described by Li et al.^27^ to activate the expression of sets of genes in an all-in-one vector (Fig. 1e). Two multiplex sgRNA expression vectors were designed based on the highest performing sgRNA for each gene identified in (Table 1). Here, each sgRNA expression cassette consists of a U6 promoter, a unique spacer sequence and an MS2 scaffold. Two distinct all-in-one multiplexed CRISPRa expression plasmids were assembled; 3TF (3 transcription factors; ERG, ETV2, FLI1) and 4EG (4 endothelial marker genes; CLDN5, CDH5, PECAM1, KDR) (Fig. 1f).

### Validation of CRISPR activation of target genes in hiPSCs

**Fig. 2:**
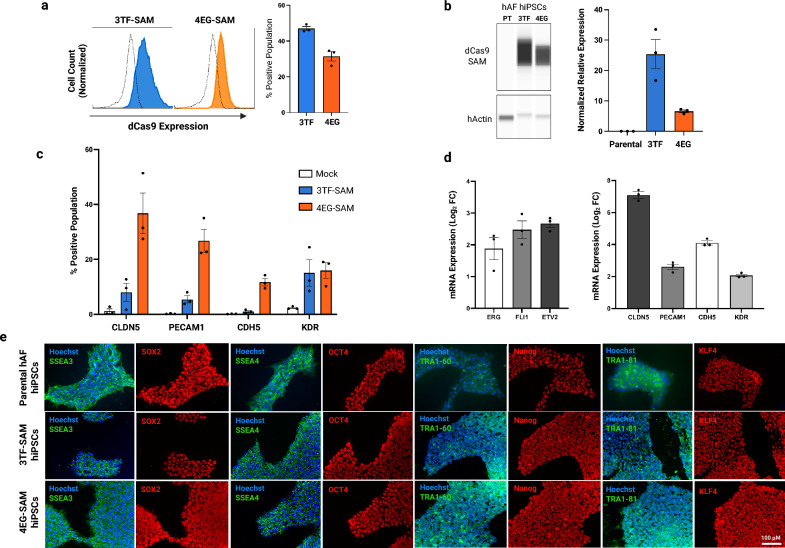
Multiplexed CRISPRa integration in hiPSCs induces core BEC genes and transcription factors. (**a**) Representative flow cytometry histograms for dCas9 expression in 3TF-SAM hiPSCs (blue), 4EG-SAM hiPSCs (orange) and parental hiPSCs (dotted). Viable cells were gated using forward scatter versus side scatter (FSC vs. SSC) followed by the gating of singlet populations (FSC-H vs. FSC-A). Bar graphs (mean ± SEM) present percentage positive population from 3 biological replicates (n = 3). (**b**) Western blot showing the expression of dCas9-SAM in CRISPRa transformed hiPSCs and parental (PT) hiPSCs. Quantification of protein bands for dCas9, normalized to hActin, showing expression levels (mean ± SEM) in 3 biological replicates (n = 3). Western blots were cropped to clearly depict the expression; uncropped image can be found in the supplementary figure S5. (**c**) Flow cytometric analysis for BEC tight junction (CLDN5, PECAM1), adherens junction (CDH5) and angiogenic protein (KDR) expression in CRISPRa transformed hiPSCs. Viable cells were gated using forward scatter versus side scatter (FSC vs. SSC) followed by gating of singlet populations (FSC-H vs. FSC-A). The bar graph (mean ± SEM) presents percentage positive population from 3 biological replicates (n = 3). (**d**) mRNA expression of transcription factors and brain endothelial genes in 3TF-SAM and 4EG-SAM hiPSCs determined by RT-qPCR. Individual Ct values were assessed in biological triplicates (n = 3) and standardized with Log_2_ fold change (mean ± SEM). (**e**) Immunofluorescence staining of parental hAF hiPSCs, 3TF-SAM hAF hiPSCs and 4EG-SAM hAF hiPSCs stained for nuclear pluripotency markers (SOX2, Oct4, Nanog, KLF4) and membrane-associated pluripotency markers (SSEA3, SSEA4, Tra1-60 and Tra1-81). Hoechst staining (blue) marks cell nuclei.

To determine if the expression of the multiplexed CRISPRa systems would have an impact on the viability of hiPSCs, human amniotic fluid (hAF) derived iPSCs (hiPSCs) were co-electroporated with a CRISPRa plasmid (3TF-SAM, 4EG-SAM or PB-SAM-DEST) and piggyBac transposase. The transfected cells underwent hygromycin selection and growth was monitored for 10 days. Stably transfected iPSCs were obtained after 2–3 days in the presence of hygromycin, with stably transfected mock CRISPRa (PB-SAM-DEST plasmid lacking an sgRNA cassette) hiPSCs exhibiting a doubling time of ~ 1.3 days (Figure S2). The doubling time of stably transfected hiPSCs with an sgRNA cassette (3TF-SAM, 4EG-SAM) did not appear to be different from mock transfected hiPSCs. Expression of dCas9-SAM was verified in the transfected hiPSCs using flow cytometry and western blotting (Fig. 2a,b). The efficacy of CRISPRa-mediated induction of the sgRNA-targeted genes was evaluated using flow cytometry for BBB-specific tight junction, adherens junction and endothelial proteins (CLDN5, PECAM1, CDH5 and KDR; Fig. 2c). We observed an induction of all four BBB-specific surface markers in hiPSCs expressing in both the 4EG-SAM and 3TF-SAM constructs, with higher levels of expression observed in 4EG-SAM cells. In contrast, mock and parental (unmodified hiPSCs) hiPSC cultures exhibited negligible expression of the targeted markers (Fig. 2c). Interestingly, targeting the ETS transcription factors (3TF-SAM) produced significant levels of induction of CLDN5, PECAM1 and KDR, and lesser induction of CDH5 expression. While it was not necessarily expected that the 3TF-SAM cells would exhibit upregulation of these specific BEC markers, their expression is consistent with the ETS factors governing iPSC differentiation into BECs. To further validate the induction of the CRISPRa-targeted genes, RT-qPCR analysis of gene expression in transfected hiPSCs against mock lines revealed levels of RNA transcript induction (Fig. 2d) that were similar to protein level induction in both transformed cell lines (Fig. 2c). Stable CRISPRa-integrated hiPSC lines were isolated and expanded for downstream analysis, and the retention of pluripotency marker expression was validated using immunofluorescence. Figure 2e shows that the pluripotency marker expression profile (SSEA3, SSEA4, SOX2, OCT4, Nanog, TRA1-60, TRA1-81 and KLF4) of the 3TF-SAM and 4EG-SAM hiPSCs was indistinguishable from the parental hiPSCs.

### Activation of 3TF or 4EG genes induces and accelerates hiPSC BEC differentiation

**Fig. 3:**
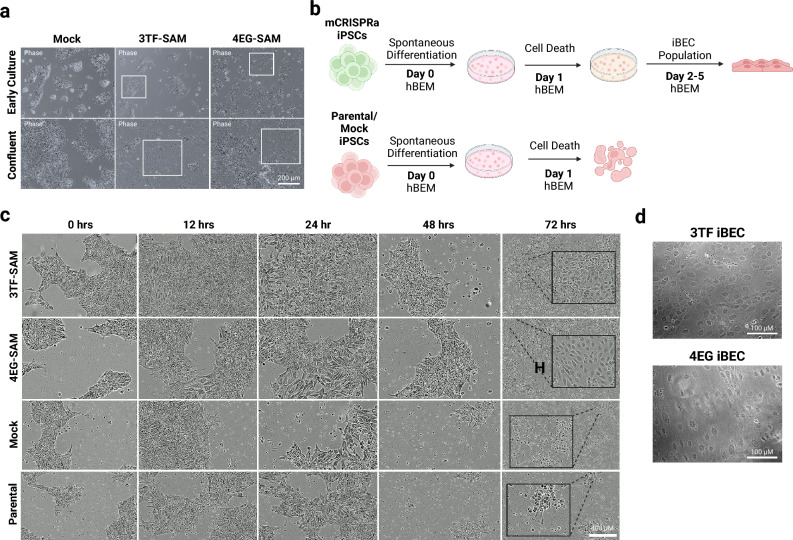
CRISPRa-mediated induction of BBB genes and transcription factors initiates directed differentiation into iBEC-like cells. (**a**) Spontaneous differentiation (boxed regions) observed in 3TF-SAM and 4EG-SAM transfected hiPSCs and normal culture conditions observed in mock transfected hiPSCs. (**b**) Schematic overview of novel differentiation scheme employed in CRISPRa-transformed hiPSCs, illustrating the culture outcomes for both parental and CRISPRa-transformed cells. (**c**) Imaging of differentiation of CRISPRa-transformed hiPSCs to iBEC-like cells over 72 h. Phase contrast images were captured at 10X every 6 h with a live cell imaging system (Incucyte). (**d**) Resulting iBEC-like cells after 5 days of differentiation in hBEM exhibit endothelial-like morphology.

Observation of the transformed hiPSCs in culture resulted in the unexpected finding that both the 3TF-SAM and 4EG-SAM hiPSC cultures exhibited consistent spontaneous differentiation (Fig. 3a). In contrast, spontaneous differentiation was not observed in mock transfected hiPSC cultures, which were indistinguishable from the parental hiPSCs.

BEC differentiation of the 3TF-SAM and 4EG-SAM cells was conducted using established protocols, as previously described^27^. Transitioning the 3TF-SAM and 4EG-SAM hiPSCs into KOEB pre-differentiation media resulted in a change to cobblestone-like morphology, consistent with endothelial cells (ECs) derived from mock and parental hiPSCs. The cells acquired a distinct, flattened elongated morphology at the end of the differentiation period in complete EM media (Figure S3). We subsequently assessed if BEC differentiation could be directly induced by culturing the 3TF-SAM and 4EG-SAM hiPSCs, as well as the parental and mock hiPSCs, in complete human BEC media (hBEM). CRISPRa-integrated hiPSC cultures that retained pluripotent markers were seeded for differentiation. As expected, all cell lines expanded and began differentiating within the first 24 h (Fig. 3b,c). Whereas complete cell death was observed for both parental and mock hiPSCs by 72 h, the 3TF-SAM and 4EG-SAM hiPSCs remained viable and continued to differentiate as observed by emerging colonies of flattened, elongated endothelial-like cells. At day 5 in hBEM, the morphology of 3TF-SAM and 4EG-SAM cells gave to confluent, cobblestone, elongated morphology (Fig. 3d). No notable differences between the cell lines were observed in terms of purity, quantity and efficiency. To further validate the reproducibility of our multiplexed CRISPRa platform, we repeated the transduction and differentiation using two additional hAF-iPSC lines, hAF09 and hAF12 (Table S2). The hAF09 and hAF12 lines showed similar accelerated differentiation and BEC morphology (Figure S4).

### Characterization of BBB phenotype induction in CRISPRa-differentiated iBECs

**Fig. 4:**
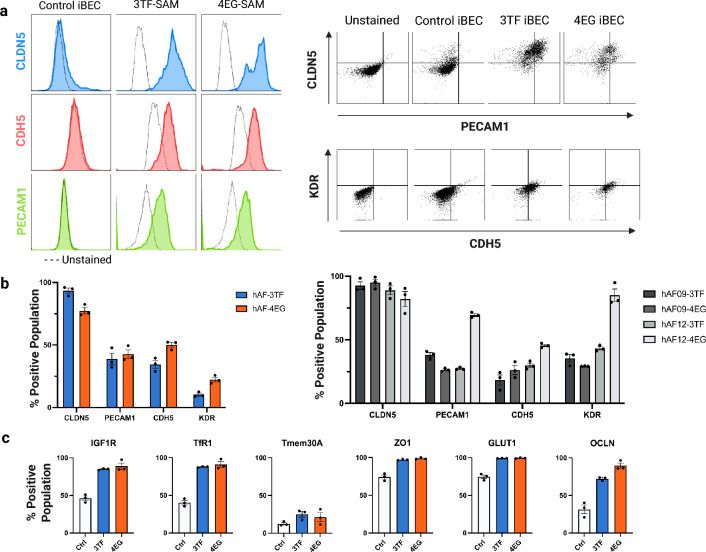
CRISPR-mediated BEC reprogramming enhances expression of BBB- and BEC-specific markers. (**a**) The left panels display flow cytometry histograms comparing the expression of CLDN5 (blue), CDH5 (red) and PECAM1 (green) in control iBECs (standard differentiation scheme) and 3TF-SAM and 4EG-SAM iBECs using the abbreviated differentiation scheme. Viable cells were gated using forward scatter versus side scatter (FSC vs. SSC) followed by singlet gating (FSC-H vs. FSC-A) to exclude aggregates. The right panels show dot plots and the corresponding gating strategy used for final marker analysis. (**b**) Flow cytometric analysis of BEC tight / adherens junction (CLDN5, PECAM1, CDH5) and angiogenic protein (KDR) expression in CRISPRa-differentiated iBECs. The left panel presents BEC marker analysis for CRISPRa-differentiated iBECs derived from hAF hiPSCs. The right panel presents replicated CRISPRa differentiation with 3TF-SAM and 4EG-SAM in two additional parental hiPSC cell line (hAF09, hAF12). Viable cells were gated as above. Bar graphs (mean ± SEM) present percentage positive from 3 biological replicates (n = 3). (**c**) Flow cytometric analysis of BBB transporters and markers in control iBECs (white) 3TF-SAM (blue) and 4EG-SAM (orange) iBECs. Viable cells were gated as above. Bar graphs (mean ± SEM) present percentage positive from 3 biological replicates (n = 3).

The CRISPRa-differentiated iBECs were further characterized to validate BEC-specific marker expression and transcriptome profile. We first assessed dCas9 expression in CRISPRa-differentiated iBECs by western blotting to confirm the absence of transgene silencing in the terminally differentiated state. The CRISPRa iBECs derived from all three hiPSC lines (hAF, hAF09 and hAF12) retained strong dCas9-SAM expression (Figure S5). Moreover, flow cytometry was performed to compare BEC surface protein expression in CRISPRa iBECs (accelerated differentiation) with iBECs differentiated using the standard protocol (Fig. 4a). The CRISPRa iBECs (both 3TF-SAM and 4EG-SAM) differentiated using the abbreviated protocol exhibited heterogeneous expression of CLDN5, PECAM1 and CDH5. While the vast majority of the cells were positive for CLDN-5, only 40–50% of the cells were positive for CDH5 and PECAM1 (Fig. 4b). In contrast, expression of the markers in parental hiPSCs differentiated using the standard protocol was largely indistinguishable from unstained control cells (Fig. 4b). The induction and expression of CLDN5, PECAM1, CDH5 and KDR in all three iPSC lines were assessed, revealing that the CRISPRa-differentiated iBECs expressed the markers regardless of the parental iPSC line of origin (Fig. 4b). Interestingly, the levels of induction were generally consistent among the three iPSC lines, with the notable exceptions of PECAM1 and KDR in hAF12-4EG, suggesting that factors affecting the efficiency of induction were relatively consistent among the iPSC lines. Furthermore, flow cytometry analysis was performed to validate the expression levels of BBB tight junction proteins (ZO1 and Occludin) and proteins involved in receptor and carrier-mediated transcytosis and transport such as IGF1R, TfR1, TMEM30A and Glut1. The data shows that the expression of these important factors for BBB function was elevated in the CRISPRa-differentiated iBEC lines relative to the control iBECs (Fig. 4c).

**Fig. 5:**
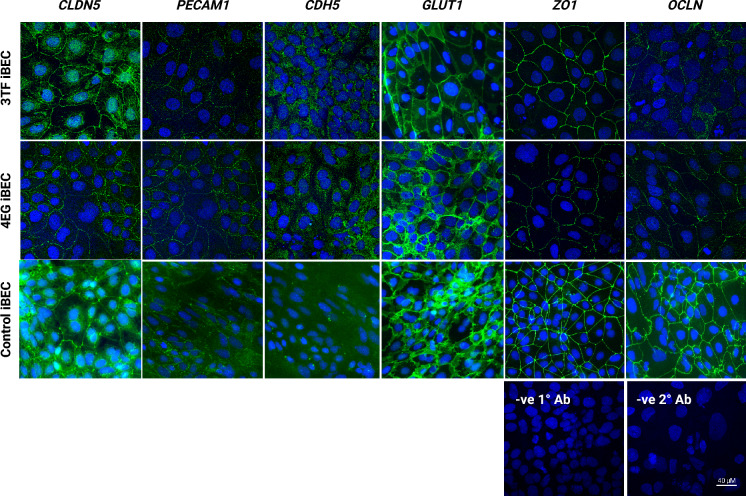
Immunofluorescence imaging of the localization and expression of brain endothelial cell markers in CRISPR-differentiated iBECs. Immunofluorescence staining of iBECs derived from hAF hiPSCs. The iBECs were generated using two protocols: The abbreviated protocol (Fig. 3b) was used for 3TF-SAM and 4EG-SAM, and the standard protocol was used for control iBEC differentiation. The brain endothelial markers evaluated include tight junction proteins (CLDN5, ZO1, OCLN), an adhesion molecule (PECAM1) and adherens junction protein (CDH5). The glucose transporter (GLUT1), characteristic of BBB cells is also shown. Hoechst33342 staining (blue) was used to counterstain the cell nuclei. All images include negative controls stained with secondary antibody (− ve 2° Ab) to confirm specificity.

The expression and subcellular localization of BEC proteins were assessed in CRISPRa-differentiated iBECs by immunocytochemistry. Figure 5 shows that CLDN5 and PECAM1 localized in the cell membrane, producing a staining pattern consistent with ZO-1 and GLUT1. In contrast to tight junction proteins, the adherens junction protein CDH5 exhibited a diffuse staining pattern in CRISPRa iBEC cells that was not limited to intracellular junctions.

**Fig. 6:**
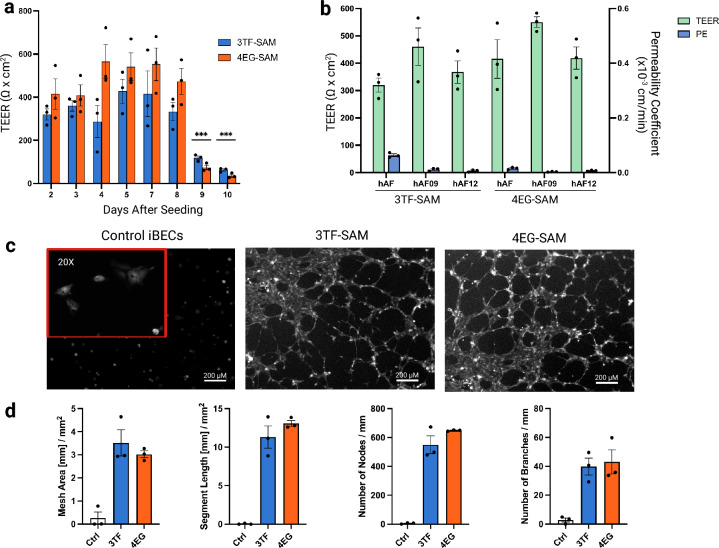
Functional characterization of barrier integrity, selective permeability and angiogenic potential in iBECs. (**a**) Transendothelial electrical resistance (TEER) measurements over a 10 day post-seeding period demonstrate the dynamics of barrier integrity in 3TF-SAM and 4EG-SAM iBECs derived from hAF hiPSCs. Stable TEER values were observed from days 2 through 8, with TEER values declining significantly on days 9 and 10. Statistical significance was determined using a two-way ANOVA with Sidak’s multiple comparison test, comparing each time point to day 2. Asterisks denote statistical significance (*** p < 0.0001). No significant differences were observed between iBECs derived from the 3TF- and 4EG-SAM iPSCs. Thus, optimal barrier characteristics are observed from approximately day 4–7 post-seeding. Bar graphs (mean ± SEM) were generated from 3 biological replicates (n = 3). (**b**) Comparison of TEER values and paracellular diffusion coefficient (P_e_) determined using a small molecule tracer sodium fluorescein (NaFl). The graph shows the formation of a tight, highly selective barrier, characterized by high TEER and low P_e_. Data includes iBECs derived from three distinct hiPSC cell lines (hAF, hAF09, hAF12). Bar graphs (mean ± SEM) were generated from three biological replicates (n = 3) (**c**) Angiogenesis assay comparing tubule formation capability of iBECs derived from the standard differentiation (Control) protocol and the abbreviated protocol (3TF-SAM and 4EG-SAM). Cells were plated on undiluted Matrigel and induced with VEGF to assess their potential to form branched, capillary-like networks. Cells were stained with CMFDA and visualized with an Axiovert 200 M microscope (Zeiss). (**d**) Quantitative metrics analyzing the results from the angiogenesis assay. The bar graphs present the structural complexity of the formed networks, including mesh area, segment length, nodes and branches for iBECs differentiated under various protocols. Analysis was performed with Angiogenesis Analyzer plugin on Fiji Software, and the data are normalized to the 10X field of view in mm. Bar graphs (mean ± SEM) were generated from 3 biological replicates (n = 3).

To assess the ability of CRISPRa iBECs to form a functional and intact barrier, the CRISPRa iBECs were seeded and cultured in semipermeable transwell inserts and transendothelial electrical resistance (TEER) was measured every 24 h to characterize barrier formation and integrity (Fig. 6a). Strong barrier formation was observed as early as day 2, while peak TEER values were observed at approximately day 4–7 (377 ± 49 Ω·cm^2^ in 3TF-SAM iBECs and 553 ± 39 Ω·cm^2^ in 4EG-SAM iBECs). TEER values for the 4EG-iBECs were distinctly greater than those observed for 3TF-iBECs. By day 8–9, TEER values declined sharply for both 3TF- and 4EG-iBECs, a trend that is consistent with our previous work on the parental iBECs^28^. We subsequently assessed sodium fluorescein (NaFl) paracellular permeability (P_e_) as a measure of barrier integrity and tight junction function. In CRISPRa iBECs, lower permeability was observed in 4EG-SAM iBECs (Pe = 0.0084 ± 0.002 × 10⁻^3^ cm/min) compared to 3TF-SAM iBECs (0.027 ± 0.091 × 10⁻^3^ cm/min), consistent with their higher TEER values (Fig. 6b). Both CRISPRa iBECs demonstrate highly selective paracellular diffusion as their P_e_ values were substantially lower than previously reported values in hcMEC/D3 cells^29^ and comparable to other hiPSC models (P_e_ = 0.028–0.034 × 10⁻^3^ cm/min)^29–31^.

The ability to undergo angiogenesis (the formation of blood vessels and capillaries) is a key characteristic of primary BECs. In isolated primary BECs, this is demonstrated as the ability to form tubules in the presence of angiogenic factors such as VEGF^30,31^. However, some iPSC-derived in vitro BBB models do not report BECs being able to undergo angiogenesis in in vitro assays. We hypothesized that inducing the expression of KDR (VEGFR2) could induce an angiogenic phenotype on iPSC-derived BECs. Figure 6c shows that both 3TF-SAM and 4EG-SAM iBECs seeded in undiluted Matrigel layers containing VEGF formed networks of tubules, which is indicative of the angiogenic potential (Fig. 6d). This is in sharp contrast to the absence of angiogenic phenotype in the standard differentiation with parental iPSCs (Fig. 6c,d).

### Transcriptomic analysis of 3TF-SAM and 4EG-SAM iBECs

**Fig. 7:**
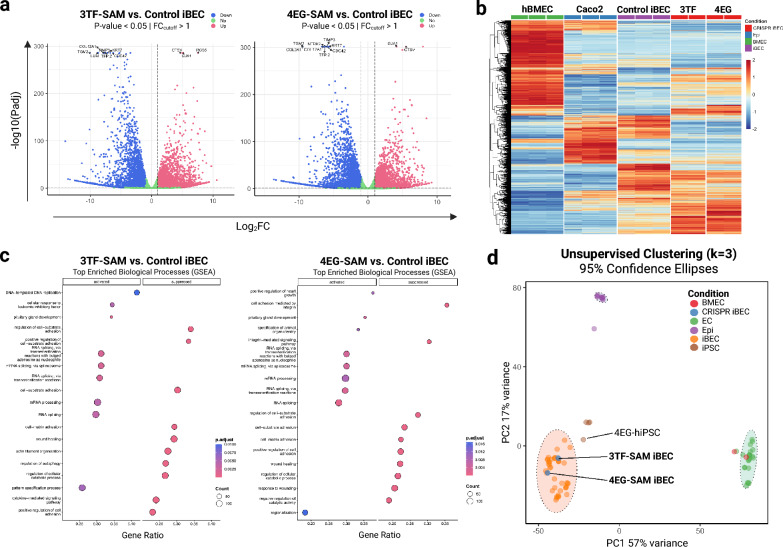
Transcriptome profiling of CRISPRa differentiated iPSC-derived brain endothelial cells. (**a**) Volcano plot highlighting differentially expressed genes between 3TF-SAM (left) and 4EG-SAM (right) iBECs compared to standard iBECs. (**b**) Heatmap of global gene expression (log_2_-transformed read counts) across control iBECs, CRISPRa-transformed iBECs, primary hBMEC and Caco2 epithelial cell lines. (**c**) Gene set enrichment analysis (GSEA) comparing CRISPRa iBECs (3TF-SAM and 4EG-SAM) to control iBECs. Significantly enriched pathways are shown based on normalized enrichment scores. (**d**) Principal component analysis (PCA) of transcriptomic profiles comparing CRISPRa iBECs to external datasets of iBECs, hBMECs, hiPSCs, endothelial cells (ECs) and epithelial cells. Unsupervised k means clustering (k = 3) was performed on the PCA coordinates to identify transcriptionally distinct cell populations. Data sources for the meta-analysis are listed in Table S8.

To investigate the global transcriptional changes associated with CRISPRa-mediated differentiation, RNA-sequencing analysis was performed on CRISPRa iBECs and iBECs derived from the standard protocol. When comparing the transcriptomic profile between 3TF-SAM and 4EG-SAM iBECs (Figure S6) we found that the majority of variation (62.5%) was attributable to differences between the cell lines, while approximately 20% variation was observed between samples. Next, we assessed CRISPRa-differentiated iBECs (abbreviated protocol) against control iBECs (standard differentiation protocol) revealing approximately 3,000 differentially expressed genes (Fig. 7a). Among the most significantly upregulated genes, both 3TF-SAM and 4EG-SAM iBECs shared robust upregulation of *GJA1* (Connexin 43) and *RGS*5 which are implicated in barrier communication and stabilization (Table S6 and S7)^32–35^. Moreover, we also observed upregulation of *CTSV,* which has been reported to be decreased in endothelial senescence, leading to *ALDH1A2* accumulation and p21 arrest^36,37^. In contrast, the epithelial marker *KRT7* (Keratin 7) was drastically suppressed in CRISPR-iBECs compared to control iBECs. Together this upregulation of barrier-specific markers and suppression of non-endothelial signatures show that CRISPR-based activation promotes unique brain endothelial lineage commitment and marker expression patterns compared to the standard protocol. While activation of these genes suggests reinforcement of brain endothelial-associated lineages, this interpretation remains preliminary and warrants further investigation into the involvement of the respective signaling pathways. Comprehensive transcriptomic analysis examining endothelial and mesodermal gene networks is necessary to confirm whether CRISPR-based activation promotes lineage commitment rather than solely on upregulation of individual genes.

To benchmark and discriminate the expression profile to bona fide primary human BEC and epithelial cells, global expression heatmaps were generated with CRISPRa-transformed iBECs, standard protocol differentiated iBECs (Control), primary human brain endothelial cells (hBMECs) and epithelial cells (Caco2) (Fig. 7b). Hierarchal clustering suggests that CRISPRa-transformed iBECs exhibit a unique transcriptional profile that retains partial overlap with control iBECs, epithelial cells and hBMECs. Moreover, important differences between the control iBECs and CRISPRa-transformed iBECs are evident, including a region of reduced epithelial expression signatures.

To gain insight into the biological pathways associated with CRISPR-mediated differentiation, gene set enrichment analysis (GSEA) was performed relative to control iBECs (Fig. 7c). In both 3TF-SAM and 4EG-SAM iBECs, strong activation of RNA processing and spliceosome pathways relative to control iBECs is observed, consistent with transcriptional remodelling. On the other hand, pathways associated with cell-matrix adhesion signalling, integrin signalling and wound responses were suppressed. These findings suggest that CRISPRa promotes an active transcriptional remodelling of cell state while reducing adhesion and stress-associated processes compared to control iBECs. This transcriptional profile may indicate a less stress-activated or less senescent cell state than that of standard-protocol-derived iBECs.

Finally, to investigate the transcriptomic identity of CRISPRa iBECs, a meta-analysis was performed to compare global gene expression profiles across a panel of related cell types, including previously published datasets: iBECs, hiPSCs, primary brain endothelial cells (hBMECs), generic primary endothelial cells (ECs) and epithelial cells (ECs) (Table S8)^20,38–43^. Principal component analysis (PCA) revealed that CRISPRa iBECs cluster closely with other iBEC populations with clear separation from epithelial cells (PC2) and hiPSCs (PC1), which is confirmed by k-means clustering (Fig. 7d). It is evident that CRISPRa iBECs continue to group closely with other iBEC models and are distinct from primary hBMEC cell lines and other generic primary endothelial cell lines. This pattern is also evident when focusing on expression heatmaps of the top 20 differentially expressed genes across all cell populations (Figure S8). A similar pattern was observed when focusing on marker genes for brain endothelial cells. Figure S9 compares the expression of 74 genes enriched in brain capillary endothelial cells and 93 genes enriched in choroid plexus cells^44^. The figure confirms that hBMECs are enriched in endothelial cell genes and depleted of choroid plexus genes relative to the other cell types. Moreover, differentiation of iPSCs to iBECs resulted in an endothelial gene expression pattern that was increasingly distant from Caco2 cells in a manner that is consistent with (Fig. 7d). Whereas Caco2 cells express less than half the choroid plexus genes, the parental and 4EG iPSCs exhibited weak expression of most choroid plexus genes. Again, iBEC differentiation resulted in a noticeable decrease in the expression of a certain clusters of choroid plexus marker genes, including CLIC6^45^. While iBEC cells exhibit a BBB phenotype, it is clear that iPSC-based static BBB models require further refinement to achieve a gene expression pattern that is fully consistent with endogenous hBMECs.

## Discussion

The integrity of the BBB is essential for protecting the brain while enabling the selective transport of nutrients and molecules vital for CNS function. As such, the development of robust and predictive in vitro BBB models is critical for advancing CNS drug screening platforms and investigating vascular contributions to neurological disorders. Here, we have described a novel transcriptional reprogramming strategy that leverages CRISPR/dCas9 to generate hiPSC-derived brain endothelial-like cells. By inducing the gene expression of key BEC markers (CLDN5, PECAM1, CDH5, KDR) or endothelial transcription factors (ETV2, ERG, FLI1) we were able to establish a directed differentiation strategy. The targets were selected partly because these endothelial-specific genes are known to be under expressed or deficient in established BEC differentiation protocols.

Here, we report that leveraging CRISPRa technology for a targeted directed differentiation strategy enables accelerated differentiation of hiPSCs into BECs that exhibit barrier properties and selective permeability, characteristic BEC morphology, and expression of requisite BBB tight junction and transporters/receptors. The CRISPRa hiPSCs appeared to be primed and responsive to differentiation cues which correlate positively with higher gene activation. In contrast, hiPSCs expressing a mock CRISPRa vector exhibited culture behavior indistinguishable from the parental cells, confirming that the differentiation-induced changes were being driven by targeted gene activation. Thus, our working hypothesis is that the CRISPRa hiPSC lines exist in a partially pre-differentiated state that enables efficient BEC differentiation upon exposure to BEC differentiation cues. This pre-induction of brain endothelial-associated transcriptional signals appeared to enhance the ability of the CRISPR-integrated hiPSCs to survive endothelial culture conditions during lineage commitment, providing a “priming” effect that facilitated the development of endothelial identity. We observed that a simplified five-day differentiation protocol successfully produced terminally differentiated and functional brain endothelial-like cells. The differentiated CRISPRa iBECs exhibited expression of key BBB tight junction, adherens junction and adhesion proteins, such as CLDN5, CDH5, PECAM1, ZO1, and Occludin, as well as BBB-enriched receptors and transporters such as TfR1, IGF1R, TMEM30A and GLUT1. Compared to conventional iBEC differentiation methods, our CRISPRa-based strategy generated cells with comparable BEC characteristics, including the formation of robust barrier achieving TEER values up to 553 Ω·cm^2^ and low NaFl paracellular diffusion rates between 0.01 and 0.12 × 10⁻^3^ cm/min. We found that 4EG-iBECs exhibited higher TEER values (553 ± 39 Ω·cm^2^) than 3TF-iBECs (377 ± 49 Ω·cm^2^). We also expected that activating KDR would help iBECs develop the ability to undergo angiogenesis. Indeed, when treated with VEGF, both 3TF-iBECs and 4EG-iBECs showed cell sprouting and branching, confirming their angiogenic potential. Thus, CRISPRa-regulation of gene expression appears to be capable of inducing innate processes, such as angiogenesis, that are currently deficient in many iBEC models. Collectively, these findings highlight the potential of utilizing synthetic biology tools to enhance BBB models.

In line with previous studies that demonstrated transgene expression of the ETS transcription factors drives BEC differentiation and improves phenotype^46–48^, we anticipated that the induction of BBB-specific transcription factors (3TF) could result in directed differentiation. However, the induction of BBB-specific tight junction genes was not expected to drive differentiation in a similar manner (Fig. 3), suggesting that the 4EG genes may play an unrecognized role in supporting the BEC differentiation process. Thus, further investigation is warranted to explore the mechanisms by which the induced tight junction and BBB genes influence BEC differentiation, particularly to the potential involvement in the KDR and the Wnt signaling pathways^49,50^. Understanding these mechanisms could provide deeper insights to improve BBB modelling and differentiation, as well as enhancing the robustness of future in vitro BBB models.

We characterized differentiated cells for BEC markers and function to assess their suitability as a BBB model. When compared to 3TF-iBECs, the expression of tight junction proteins (CLDN5, PECAM1) and an adherens junction protein (CDH5) was observed to be higher when directly targeted by CRISPRa in the 4EG-iBECs (Fig. 2C). This suggests that directly targeting genes with CRISPRa yields greater induction than indirect targeting through transcription factors for downstream expression^51–53^. Moreover, it is not clear if the level of transcription factor activation in the 3TF-iBECs would be sufficient to activate all BEC-relevant downstream genes. Thus, consideration should be given to the activation strategy (direct or indirect) utilized along with sgRNA efficiency to select an appropriate outcome for individual genes in future multiplexed CRISPR reprogramming systems.

A persistent challenge found in iPSC-derived BBB models is lineage heterogeneity, where analysis of iBEC gene expression reveals both epithelial characteristics and a failure to fully exit the premature state. The accelerated and optimized differentiation strategy leveraging CRISPR/Cas9 technology described herein also fails to fully replicate the authentic BEC phenotype and lineage. This is supported by the transcriptomic analysis observed in the PCA plot in (Fig. 7d) where CRISPRa-differentiated iBECs still cluster with other iBEC models. Moreover, the global expression heatmap (Fig. 7b) demonstrates that CRISPRa iBECs also exhibit a transcriptomic pattern that partially overlaps with control iBEC populations despite the suppression of a subset of epithelial (Caco2) genes (e.g., *KRT7* downregulation). This suggests that CRISPRa transformed iBEC expression profiles remain consistent with the standard protocol which is consistent with the PCA analysis (Fig. 7d) and the Pearson correlation coefficient similarity matrix (Figure S7). One possible explanation is that, despite improvements in marker expression, CRISPR-differentiated iBECs share similar limitations inherent to in vitro hiPSC differentiation systems. These may include incomplete maturation, absence of key in vivo microenvironmental cues (such as interactions with pericytes, astrocytes, and neural cells), and limited exposure to physiological mechanical forces like shear stress. As a result, the transcriptomic profiles remain highly similar across iBEC models but still diverge from bona fide human BECs, which develop within a complex, dynamic vascular niche in vivo. Similarly, heatmaps based on canonical brain endothelial and choroid plexus genes^44^ (Figure S9) further emphasize the distinct hybrid state of iBECs relative to gene signatures derived from endogenous cells. Moreover, it appears that differentiation toward iBECs does not produce a discernible trajectory toward a relevant epithelial cell lineage (choroid plexus). This indicates that, beyond targeted gene activation, incorporating additional biological and environmental cues, such as interactions with glia and neurons, extracellular matrix components, and physiological mechanical forces, may be essential to fully replicate the complex transcriptional programs and functional characteristics of bona fide BECs. Thus, while the directed reprogramming strategy described in this study achieved a BEC phenotype, there is a clear need for further improvements. To highlight this, further analysis of our RNA-seq datasets was performed on raw counts (normalized to transcripts per million, TPM) and key markers were extracted for comparison (Figure S10). At the differentiated iBEC state, both 3TF-SAM and 4EG-SAM populations revealed low to negligible expression of most induced targets, with the exception of CLDN5 in 4EG-SAM and ERG in both 3TF-SAM and 4EG-SAM iBECs. Notably, 4EG-SAM hiPSCs demonstrated upregulation of all four target transcripts at the pluripotent stage, indicating that the CRISPRa constructs were functional prior to differentiation. The BBB-associated transporters and surface markers (GLUT1, IGR1R, TfR1, ZO1 and TMEM30A) were readily detected in both CRISPRa iBEC populations confirming that the differentiation protocol induced major BBB identity features. Notably, the epithelial marker EPCAM was robustly expressed at both the iPSC stage and in iBECs (Figure S10), indicating that iPSCs appear to exhibit a pre-existing epithelial characteristic that is retained following differentiation. This phenomenon is also observed in the heatmap of choroid plexus genes (Figure S9B). Despite the persistent dCas9/SAM expression at both the hiPSC and differentiated state, we observed a failure to sustain activation of endogenous targets at the terminal iBEC stage indicating that transcriptional reprogramming barriers emerge as a result of differentiation. We propose that chromatin compaction and associated remodeling complexes may restrict accessibility of key loci in differentiating cells, limiting CRISPRa-driven activation during late-stage reprogramming and maturation. Therefore, future work should investigate epigenetic co-activation strategies, including chromatin-modifying CRISPR effectors such as CRISPRon/CRISPRoff^54^, or small molecule epigenetic modulators to overcome potential silencing mechanisms and improve the robustness of endogenous gene activation platforms for terminal iBEC differentiation.

Interestingly, when comparing the top differentially expressed genes between CRISPRa iBECs and control iBECs, we identified several key genes that were upregulated and relevant to BBB fidelity and commitment. For example, a recent study demonstrated that *RGS5* is a marker of BBB integrity and neuroprotection in focal cerebral ischemia^34^. The loss of *RGS5* leads to endothelial dysfunction, tight junction protein relocalization and increased BBB permeability. Furthermore, *GJA1* (Connexin 43) was highly upregulated compared to control iBECs. Recent works by Krolak et al. demonstrated that endothelial gap junctions are required for neurovascular coupling that coordinates blood flow with neuronal activity^33^. Restoring *GJA1,* comparable to hBMECs, demonstrated that cells were hemodynamically competent and elucidated its role in the distribution of resources. Beyond its role in electrical signaling, another study revealed a structural role in which it interacts with the tight junction protein ZO-1 to stabilize the barrier complex^35^. Disruption of *GJA1* revealed dysfunction in CLDN5 and ZO-1 formation, resulting in hyperpermeability. Therefore, the robust expression in this marker reveals that CRISPR iBECs exhibit sound electrophysiological and structural factors for the BBB phenotype. In summary, the 3TF and 4EG reprogramming strategies do not solely address barrier tightness but may also have an implicit impact on gene networks regulating brain endothelial-specific identity and function by silencing aberrant epithelial markers (e.g., *KRT7*) and enhancing machinery for neurovascular coupling and junction formation (e.g., *GJA1 and RGS5*).

Future modifications to this multiplexed gene regulation system could implement CRISPR interference (CRISPRi) to target epithelial genes and other genes not expressed in endogenous BECs. Additionally, recent studies identifying transcription factors and genes not previously associated with BEC differentiation and function should be incorporated into future CRISPRa/i system designs^51^. Moreover, additional phenotypic deficiencies observed in transcriptomic analysis (Fig. 7C), such as immune cell interactions and transient barrier properties, could be enabled by the expression of key functional genes and the utilization of more representative multicellular environments with flow characteristics. Experiments exploring the effect of shear stress and more complex multicellular microphysiological systems on iBEC phenotype will continue to improving BEC differentiation protocols^55^. Ultimately, we expect that phenotype refinement of in vitro models will involve addressing the various limitations imposed by static 2D monoculture systems, with multiplexed gene regulation playing an important role in fully realizing the vast potential of iPSC-derived cell and tissue models.

A key limitation of this study is the variability and potential genotoxicity associated with piggyBac transposase-mediated genomic integration of CRISPRa constructs into hiPSCs^56^. The genomic integration site for PiggyBac transposase is the TTAA motif, which is widespread throughout the human genome, with reports indicating hundreds of genomic integration sites^57^. Consequently, PiggyBac integration does not permit precise control of the integration site or copy number, leading to heterogeneous transgene expression, variable gene induction and increased cell–cell variability. To address these limitations, future iterations of this platform should incorporate site-specific integration strategies into characterized genomic safe harbors (e.g., AAVS1, CLYBL, CCR5, Rosa26, Rogi1, Rogi2) to achieve uniform expression, controlled copy number and the generation of clonally defined hiPSC lines. In parallel non-integrating approaches such as mRNA-based CRISPR systems or episomal vectors should be explored to reduce genotoxic risk while maintaining tunable transgene expression. The development of inducible CRISPRa platforms would further provide temporal control over transcriptional activation, enhancing the flexibility and scalability of directed differentiation workflows. Lastly, differentiation consistency and phenotypic characterizations in this study were limited to hiPSCs derived from human amniotic fluid. Expanding this approach to other donor cell types, such as fibroblast and bone marrow-derived cells will be critical for assessing robustness and donor-dependent variability in our CRISPR-mediated brain endothelial cell differentiation scheme.

CRISPR/Cas9 technology offers a novel and versatile method to differentiate hiPSCs through transcriptional reprogramming by targeting key genes and transcription factors. CRISPR-differentiated iBECs demonstrate functional tight junction formation as evidenced by high TEER and low paracellular permeability rates, while also exhibiting angiogenic potential. Moreover, these iBECs expressed essential BEC markers, including markers of receptor-mediated transport. Synthetic biology tools offer immense promise in the development of innovative differentiation schemes as they represent a versatile platform for accelerating, refining and fine-tuning the direct reprogramming of hiPSCs. In conclusion, CRISPR-differentiated iBECs developed in this study represent promising candidates for in vitro BBB modeling, utilizing transcriptional reprogramming as a novel approach for stem cell-based engineering.

## Materials and methods

### Vector construction

Sense and anti-sense of individual sgRNAs targeting genes for brain endothelial differentiation containing BbsI overhangs was synthesized and cloned into PB-sgRNA (MS2) (a gift from Ying Liu, Addgene plasmid # 102560) through Golden-Gate assembly. Sequence information of the validated sgRNAs of the 3-brain endothelial cell transcription factors and 4 BEC genes is listed in Table S1. To assemble arrays of multiple sgRNAs (tracrRNA and crRNA) driven by U6 promoters, each fragment was individually amplified from PB-sgRNA (MS2) containing overhangs and cloned into pGGA-Select plasmid (NEB # N0309AAVIAL) with NEBridge Ligase assembly (NEB #E1602). Three combinations of sgRNA donor plasmids were generated denoted as 4EG-Select, 3TF-Select and 4TF-CG-Select containing compatible attL1 and attL2 sites. To generate a multiplexed CRISPRa plasmid targeting key genes involved in brain endothelial differentiation (4EG-SAM, 3TF-SAM), multiplexed sgRNA fragments were amplified from 4EG-Select, 3TF-Select and 4TF-CG-Select and LR Cloned (Thermofisher #12538120) into PB-SAM-DEST (a gift from Ying Liu, Addgene plasmid # 102563) a destination vector containing piggyBac long terminal repeats (LTRs), dCas9-VP64 and MSPH SAM components. All inserts were validated through PCR and sequencing. Primer sequences used for validation are listed in Table S3.

### Cell culture

HeLa cells were obtained commercially from ATCC and cultured in HeLa Complete medium comprised of Dulbecco’s Modified Eagle Medium (Wisent, #319-005 CL) supplemented with 10% fetal bovine serum, and 1% penicillin/streptomycin. HeLa cells were passaged every 3–4 days with 0.025% trypsin–EDTA (Invitrogen, #25200056). hAF, hAF09 and hAF12 iPSC cell lines were generated in-house at the National Research Council of Canada reprogrammed from human amniotic fluid samples from patient donors. All experimental protocols using AF cells were performed following the guidelines established and approved by the National Research Council Canada Research Ethics Board. hiPSCs were cultured in mTESR1 media (StemCell Technologies #85850) and passaged every 3–4 days with an enzyme free reagent ReLeSR (StemCell Technologies #100-0483). All iPSCs were cultured on pre-coated hESC-qualified Matrix Matrigel (Corning #354277).

### Generation of HeLa-SAM and hiPSC-hAF-SAM stable cell lines

The generation of HeLa-SAM, HeLa-4EG-SAM and HeLa-3TF-SAM cell lines was accomplished a previously described protocol by Li et al. by co-transfecting 1 μg of CRISPRa-containing plasmid (SAM, 4EG-SAM, 3TF-SAM) and 0.2 μg of Super PiggyBac transposase expression vector (Systems Biosciences #PB210PA-1) in (2 × 10^5^ cells in one 96-well nucleofector plate). All HeLa cell lines were electroporated using a 4D-Nucleofector paired with SE Cell Line Kit (#V5SC-1010) and plated on a 96-well plate containing HeLa Complete medium for 48 h. Subsequently, recovered cell lines were cultured and selected for 2 weeks in HeLa-SAM media (HeLa Complete medium containing 15 μg/mL of blasticidin and 200 μg/mL of hygromycin). The expression of HeLa-SAM cell lines was examined by performing rt-qPCR and flow cytometry.

Similarly, to generate hAF-SAM cell lines (hAF-4EG-SAM and hAF-3TF-SAM, 1 × 10^6^ hAF-hiPSCs were co-transfected with 1.5 μg of the respective SAM plasmids with 0.2 μg of Super PiggyBac transposase expression vector (Systems Bioscience, #PB210PA-1). Electroporation was carried out using Nucleofector P3 Primary Cell Kit (Lonza #V4LP-3002) in a nucleocuvette. 48 h post-nucleofection, iPSCs were cultured with mTESR1-SAM media (containing 15 μg/mL of blasticidin and 200 μg/mL of hygromycin). The expression of hAF-SAM cell lines was examined by performing RT-qPCR, flow cytometry and immunofluorescence staining.

### Transactivation of HeLa-SAM cell lines

HeLa-SAM cell lines do not contain any sgRNA expressing cassettes, thus, to individually assess for sgRNA performances, (refer to Table S1) sgRNA expressing cassettes were transiently transfected through electroporation using SE Cell Line Kit with 0.5 μg of sgRNA-PB-sgRNA (MS2) plasmid as described by Li et al.^27^ 48 h post-nucleofection, expression of target genes was evaluated by rt-qPCR to individually assess sgRNA performance.

### RT-qPCR

RNA and cDNA were extracted, and reverse transcribed from HeLa-SAM cells using FastLane Cell cDNA Kit (Qiagen, #215011). Quantitative reverse transcription PCR was performed using QuantiTect SYBR Green PCR kit (Qiagen, #204141) with Bio-Rad CFX96.Relative gene expression fold change was quantified using actin as an internal control with the ΔΔCt method. Primer sequences are listed in Table S3.

### Flow cytometry

HeLa cell lines were prepared for staining using staining buffer (1 X PBS, 2% FBS, 1 mM EDTA, 0.1% sodium azide) and fixed with 10% formalin (Fisher Scientific, #SF100-4). For the detection of intracellular proteins, fixed cells were permeabilized with 0.2% TritonX (Sigma, #P7949). Cells were stained with fluorochrome-conjugated monoclonal antibodies or monoclonal primary antibodies and respective fluorochrome conjugated secondary antibody following manufacturer recommended dilutions. For each acquisition, 10,000 events were recorded on BDAccuri C6 Plus Flow Cytometer and BD LSRFortessa Cell Analyzer (BD Biosciences). All data was analyzed with FlowJo software.

Similarly, iPSC cell lines were subjugated to 10 μM of Y-27632 Dihydrochloride ROCK inhibitor (StemCell Technologies, #72302) at least 1 h prior to analysis in liquid culture. iPSCs were fixed with 10% formalin (Fisher Scientific, #SF100-4) and prepared with staining buffer (1 X PBS, 2% FBS, 1 mM EDTA, 0.1% sodium azide). Cells were stained with fluorochrome-conjugated monoclonal antibodies or monoclonal primary antibodies and respective fluorochrome conjugated secondary antibody following manufacturer recommended dilutions. For each acquisition, 10,000 events were recorded on BDAccuri C6 plus flow cytometer and BD LSRFortessa cell analyzer (BD Biosciences). Flow cytometry results were analyzed using FlowJo™ 10.10 Software (BD Life Sciences).

### Immunocytochemistry

hAF iPSCs and CRISPR-differentiated cell lines were cultured in 12-well plates on 15 mm round coverslips (Chemglass, #CLS-1760) coated with Matrigel (Corning #354277). Cells were fixed and permeabilized with ice cold methanol (Fisher Scientific, #A412P-4) for 10 min. All samples were blocked with DAKO protein block serum free solution (Agilent, #X0909) for 1 h. Cells were incubated with primary antibodies in DAKO Antibody Diluent (Agilent, # S0809) for 1 h and subsequently incubated with secondary antibodies for another hour described in Table S4. All coverslips were washed 3 times with 1 X PBS (Wisent, #311-013 CL) in between and after antibody staining. 10 μg/mL of Hoeschst33342 (Invitrogen, # H3570) was added to counterstain the nucleus and was incubated for 10 min. All cell samples were mounted in DAKO fluorescent mounting medium (Agilent, # S3023) and visualized with Axiovert 200 M Microscope (Zeiss).

### Brain endothelial cell differentiation

For the standard BEC differentiation protocol, approximately 100,000 iPSCs were seeded on Matrigel (Corning #354277) pre-coated 6-well plates containing mTESR1 and 10 μM of Y-27632 Dihydrochloride ROCK inhibitor (StemCell Technologies, #72302). Cells were cultured for 3 days or until 80% confluency was reached. Cells were treated and cultured in KOEB pre-differentiation media (KO DMEM-F12, KO serum, Glutamax supplement, MEM NEAA, B-merc) (Thermofisher, #10829018, #10828028, #35050061, #11140050, #21985-023) for 6 days. Subsequently, the media was changed and cultured in complete EM media (Human endothelial SFM media, 1% FBS, 20 ng/mL bFGF, 10 µM retinoic acid) (Thermofisher, # 11111044, HyClone, # SH30396, Fisher-Life Technologies, # 13256029, Sigma, # R2625) for two days. The cells were dissociated with accutase (Stem Cell Technologies, #7922) and seeded at a density of 1 × 10^6^ cells on collagen/fibronectin pre-coated 1.0 μm transwell inserts in 12-well companion plates with polyethylene terephthalate (PET) membranes. Cells were cultured in complete EM media containing Y-27632 Dihydrochloride ROCK inhibitor (StemCell Technologies, #72302) on the first day and regular complete EM media the second day in the apical layer of the transwell insert. The cells were subjected to analysis on the third day.

For the abbreviated BEC differentiation protocol, approximately 100,000 hiPSCs (Parental, Mock, 3TF-SAM and 4EG-SAM) were seeded on Matrigel coated plates containing mTESR1. Cells are cultured until 50% confluency and the media was changed to primary human brain endothelial media (CelProgen #M36201-01) containing 2% FBS and antibiotics (15 μg/mL of blasticidin and 200 μg/mL of hygromycin). After the fifth day of differentiation, the cells were dissociated with accutase and seeded on collagen/fibronectin pre-coated plates for further analysis.

### Transendothelial electrical resistance (TEER) measurements

TEER measurements were performed on confluent monolayer of iBECs in 12-well inserts, 24 h following media change. TEER values were obtained with CellZscope apparatus (Nanoanalytics) with 1 cm diameter electrodes and standard spectrum settings: frequency 1 Hz–100 kHz, points per decade 9 and logarithmic spacing. Empty transwell inserts were used as controls to subtract from TEER values of iBECs to obtain final TEER in Ωcm^2^.

### Sodium fluorescein permeability transport assay

Inserts containing confluent monolayer of iBECs were washed twice in PBS (Wisent, #311-013 CL) and transferred to 12-well companion plates containing pre-warmed 1X transport buffer (5 mM MgCl_2_, 10 mM HEPES, HBSS pH 7.4). 500 μL of media was removed and replaced with 500 μL of sodium fluorescein solution (50 μg/mL) for a final concentration of 25 μg/mL in each well. Inserts containing no iBECs were used as controls. 100 μL of transport buffer at the bottom of the well was aliquoted in a 96-well plate and fresh 1 X transport buffer was added to the companion plate. iBECs were incubated at 37 °C, 5% CO_2_ with gentle rotation (20 rpm) in 311DS Labnet Rotating Incubator containing orbital shaking platform. 100 μL of transport buffer at the bottom of the companion plates were collected and replaced every 15 min for up to 1 h and transferred to a 96-well plate. Sodium fluorescein standard curves were generated by formulating NaFl-1X transport buffer solutions at varying concentrations (0, 12.5, 25, 50, 100 and 200 μg/mL). Quantification of sodium fluorescein was performed using Fluostar Optima microplate reader (BMG Labtech). The permeability coefficient (Pe) calculations and formulas are described in supplementary methods.

### Western blot

Cells were washed twice with 1X PBS (Wisent, #311-013 CL) and lifted by manual scraping. Subsequently, they were pelleted at 300 × g for 5 min and stored at −80 °C until further processing. Cells were lysed with 1X RIPA buffer with protease inhibitors (BioRad, Sigma #20-188, Roche Diagnostics, #11697498001) and a Quantipro BCA assay (Sigma, #QPBCA) was performed to quantify the protein concentration for each sample. Western blot assays were performed using the Wes System (Protein Simple, #004-600) and samples were diluted to 1.2 mg/mL in sample buffer and a fluorescent master mix according to the manufacturer’s protocol (Protein Simple, # PS-ST03, #042-195). All samples, blocking reagent, primary antibodies, HRP-conjugated secondary antibodies, and chemiluminescent substrate solutions were pipetted into a plate separation module (Protein Simple, # 042-203, #042-414, #043-31, #043-379, #SM-W004-1, #SM-W008-1). Electropherograms were generated and the area under the curve (AUC) for chemiluminescent signal peaks was determined. The data and the system parameters were analyzed using Protein Simple’s Compass software. Signal intensity of each band was normalized to actin (Sigma, #A3854) to account for loading differences.

### Angiogenesis assay

A 24-well plate was coated with 500 µL of fresh undiluted Corning Matrigel (Corning #354277) and incubated at room temperature for 1 h. Approximately, 100,000 iBECs were seeded per each well suspended in 500 µL of endothelial media containing 10 µM of ROCK inhibitor (StemCell Technologies, #72302). For one well, 50 ng/mL of VEGF-165 (R&D System # 293-VE) was added to stimulate angiogenesis. Cells were incubated at 37 °C overnight. Subsequently, cells were stained with 1 µM of CellTracker Green CMFDA dye (Thermofisher #C2925) for 1 h at 37 °C before visualization.

### RNA sequencing

Cultured cells were isolated in a microcentrifuge tube spun at 300 × g for 5 min and subsequently washed with 1 X PBS. Total RNA was extracted using the Direct-zol RNA Miniprep Kit (Zymo Research, Cat. No. R2050) according to the manufacturer’s protocol. Briefly, cell pellets were lysed directly in TRI reagent, mixed with an equal volume of ethanol and the mixture was transferred to a Zymo-Spin column for binding. Columns were washed with RNA Wash Buffer, treated with DNase I, washed with RNA PreWash, washed with RNA Wash Buffer and RNA was eluted in DNase/RNase-free water. The quality and quantity of the extracted RNA were assessed using an Agilent Bioanalyzer.

RNA-seq libraries were prepared using the NEBNext Ultra II Directional RNA Library Prep Kit (Cat. No. E7760) following manufacturer’s instructions for sequencing on the Illumina NextSeq 500/550 Mid Output Kit v2.5 (150 cycles; Cat. No. 20024904). Briefly, poly(A) + mRNA was enriched using oligo-dT beads, followed by fragmentation and cDNA synthesis. End repair, dA-tailing, adapter ligation and USER-enzyme digestion were performed. Lastly, ligated cDNA fragments were amplified by PCR. Final libraries were quantified using a Qubit fluorometer (Thermo Fisher Scientific), qPCR using NEBNext Library Quant Kit (E7630) and assessed for quality using an Agilent Bioanalyzer.

Sequencing was performed on the Illumina NextSeq 550 platform using paired end (2 × 75 bp) reads. Raw sequence data were demultiplexed and converted to FASTQ format using Illumina bcl2fastq software. AdaptorRemoval was used to identify adaptor sequences and FASTX-Toolkit was used for trims and filters. Alignments were performed using STAR 2.7.11a to GRCH38.p14 and RSEM v1.3.3 was used for TPM. Data was normalized with DESeq2 and GSEA was used for gene enrichment analysis.

### Data analysis

Differential gene expression (DGE) analysis was performed on raw counts using the DESeq2 package in R^58^. Standard DESeq2 workflow was executed including size factor estimation for normalization, dispersion estimation and model fitting. Differential expression statistics were determined using the Wald test. P-values were adjusted for multiple testing using the Benjamini–Hochberg protocol to control for false discovery rate (FDR). Significantly differentially expressed genes were defined by adjusted p-values (Padj) < 0.05 and an absolute Log_2_ fold change of > 1.0. Gene annotation was performed using the AnnotationDbi package to map HGNC gene symbols^59^. Visualization was performed using the EnhancedVolcano package^60^. To identify functionally enriched biological pathways Gene Set Enrichment Analysis (GSEA) was performed using the clusterProfiler package^61^. All GSEA visualizations were generated using the enrichplot package^62^. A Pearson coefficient ($r$) was calculated between transcription profiles of all sample pairs using the standard formula and visualized with the pheatmap package:

$$r= \frac{\sqrt{\mathrm{n}\sum \mathrm{xy}-(\sum \mathrm{x})(\sum \mathrm{y})}}{\sqrt{\left[n\sum {x}^{2}-(\sum x{)}^{2}][n\sum {y}^{2}-(\sum y{)}^{2}\right]}}$$

## Supplementary Information

Supplementary Information 1.

Supplementary Information 2.

Supplementary Information 3.

Supplementary Information 4.

Supplementary Information 5.

## Data Availability

All plasmids generated in this study will be available on Addgene.org. RNA sequencing data supporting the findings of this study have been deposited in the Gene Expression Omnibus (GEO) under accession number GSE299978. Additional supporting data and other data generated during this study are included in the published article and its Supplementary Information files.

## References

[1] Chen, Y., He, Y., Han, J., Wei, W. & Chen, F. Blood-brain barrier dysfunction and Alzheimer’s disease: Associations, pathogenic mechanisms, and therapeutic potential. *Front. Aging Neurosci.* **15**, 1258640 (2023). 38020775 10.3389/fnagi.2023.1258640 PMC10679748

[2] Lau, K., Kotzur, R. & Richter, F. Blood-brain barrier alterations and their impact on Parkinson’s disease pathogenesis and therapy. *Transl. Neurodegener.* **13**, 37 (2024). 39075566 10.1186/s40035-024-00430-z PMC11285262

[3] Wu, D. et al. The blood-brain barrier: structure, regulation, and drug delivery. *Signal Transduct. Target. Ther.* **8**, 217 (2023). 37231000 10.1038/s41392-023-01481-w PMC10212980

[4] Langen, U. H., Ayloo, S. & Gu, C. Development and cell biology of the blood-brain barrier. *Annu. Rev. Cell Dev. Biol.* **35**, 591–613 (2019). 31299172 10.1146/annurev-cellbio-100617-062608 PMC8934576

[5] Sweeney, M. D., Sagare, A. P. & Zlokovic, B. V. Blood-brain barrier breakdown in Alzheimer disease and other neurodegenerative disorders. *Nat. Rev. Neurol.* **14**, 133–150 (2018). 29377008 10.1038/nrneurol.2017.188 PMC5829048

[6] Ruck, T., Bittner, S. & Meuth, S. G. Blood-brain barrier modeling: Challenges and perspectives. *Neural Regen. Res.* **10**, 889–891 (2015). 26199600 10.4103/1673-5374.158342 PMC4498345

[7] Yan, L., Moriarty, R. A. & Stroka, K. M. Recent progress and new challenges in modeling of human pluripotent stem cell-derived blood-brain barrier. *Theranostics* **11**, 10148–10170 (2021). 34815809 10.7150/thno.63195 PMC8581424

[8] Bernas, M. J. et al. Establishment of primary cultures of human brain microvascular endothelial cells to provide an in vitro cellular model of the blood-brain barrier. *Nat. Protoc.* **5**, 1265–1272 (2010). 20595955 10.1038/nprot.2010.76 PMC3109429

[9] Weksler, B., Romero, I. A. & Couraud, P.-O. The hCMEC/D3 cell line as a model of the human blood brain barrier. *Fluids Barriers CNS* **10**, 16 (2013). 23531482 10.1186/2045-8118-10-16 PMC3623852

[10] Eigenmann, D. E. et al. Comparative study of four immortalized human brain capillary endothelial cell lines, hCMEC/D3, hBMEC, TY10, and BB19, and optimization of culture conditions, for an in vitro blood-brain barrier model for drug permeability studies. *Fluids Barriers CNS* **10**, 33 (2013). 24262108 10.1186/2045-8118-10-33 PMC4176484

[11] Winkler, E. A. et al. GLUT1 reductions exacerbate Alzheimer’s disease vasculo-neuronal dysfunction and degeneration. *Nat. Neurosci.* **18**, 521–530 (2015). 25730668 10.1038/nn.3966 PMC4734893

[12] Niwa, K., Kazama, K., Younkin, S. G., Carlson, G. A. & Iadecola, C. Alterations in cerebral blood flow and glucose utilization in mice overexpressing the amyloid precursor protein. *Neurobiol. Dis.* **9**, 61–68 (2002). 11848685 10.1006/nbdi.2001.0460

[13] Urich, E., Lazic, S. E., Molnos, J., Wells, I. & Freskgård, P.-O. Transcriptional profiling of human brain endothelial cells reveals key properties crucial for predictive in vitro blood-brain barrier models. *PLoS ONE* **7**, e38149 (2012). 22675443 10.1371/journal.pone.0038149 PMC3364980

[14] O’Brown, N. M., Pfau, S. J. & Gu, C. Bridging barriers: A comparative look at the blood-brain barrier across organisms. *Genes Dev.* **32**, 466–478 (2018). 29692355 10.1101/gad.309823.117 PMC5959231

[15] Syvänen, S. et al. Species differences in blood-brain barrier transport of three positron emission tomography radioligands with emphasis on P-glycoprotein transport. *Drug Metab. Dispos.* **37**, 635–643 (2009). 19047468 10.1124/dmd.108.024745

[16] Lippmann, E. S. et al. Derivation of blood-brain barrier endothelial cells from human pluripotent stem cells. *Nat. Biotechnol.* **30**, 783–791 (2012). 22729031 10.1038/nbt.2247 PMC3467331

[17] Vatine, G. D. et al. Human iPSC-derived blood-brain barrier chips enable disease modeling and personalized medicine applications. *Cell Stem Cell* **24**, 995-1005.e6 (2019). 31173718 10.1016/j.stem.2019.05.011

[18] Foreman, K. L., Shusta, E. V. & Palecek, S. P. Defined differentiation of human pluripotent stem cells to brain microvascular endothelial-like cells for modeling the blood-brain barrier. *Methods Mol. Biol.* **2683**, 113–133 (2023). 37300771 10.1007/978-1-0716-3287-1_10 PMC10389759

[19] Linville, R. M. et al. Human iPSC-derived blood-brain barrier microvessels: Validation of barrier function and endothelial cell behavior. *Biomaterials* **190–191**, 24–37 (2019). 30391800 10.1016/j.biomaterials.2018.10.023 PMC6289621

[20] Sandler, V. M. et al. Reprogramming human endothelial cells to haematopoietic cells requires vascular induction. *Nature* **511**, 312–318 (2014). 25030167 10.1038/nature13547 PMC4159670

[21] Bryant, A. et al. Endothelial cells are heterogeneous in different brain regions and are dramatically altered in Alzheimer’s Disease. *J. Neurosci.* **43**, 4541–4557 (2023). 37208174 10.1523/JNEUROSCI.0237-23.2023 PMC10278684

[22] Lu, T. M. et al. Human induced pluripotent stem cell-derived brain endothelial cells: Current controversies. *Front. Physiol.* **12**, 642812 (2021). 33868008 10.3389/fphys.2021.642812 PMC8044318

[23] Lu, T. M. et al. Pluripotent stem cell-derived epithelium misidentified as brain microvascular endothelium requires ETS factors to acquire vascular fate. *Proc. Natl. Acad. Sci. USA* **118**, e2016950118 (2021). 33542154 10.1073/pnas.2016950118 PMC7923590

[24] Charlebois, C. et al. Development of a Blood-brain barrier permeability assay using human induced pluripotent stem cell derived brain endothelial cells. *Methods Mol. Biol.* **2454**, 397–410 (2022). 33881753 10.1007/7651_2021_393

[25] Delsing, L. et al. Barrier properties and transcriptome expression in human iPSC-derived models of the blood-brain barrier. *Stem Cells* **36**, 1816–1827 (2018). 30171748 10.1002/stem.2908

[26] Konermann, S. et al. Genome-scale transcriptional activation by an engineered CRISPR-Cas9 complex. *Nature* **517**, 583–588 (2015). 25494202 10.1038/nature14136 PMC4420636

[27] Li, S., Zhang, A., Xue, H., Li, D. & Liu, Y. One-step piggyBac transposon-based CRISPR/Cas9 activation of multiple genes. *Mol. Ther. Nucleic Acids* **8**, 64–76 (2017). 28918057 10.1016/j.omtn.2017.06.007 PMC5485764

[28] Ribecco-Lutkiewicz, M. et al. A novel human induced pluripotent stem cell blood-brain barrier model: Applicability to study antibody-triggered receptor-mediated transcytosis. *Sci. Rep.* **8**, 1873 (2018). 29382846 10.1038/s41598-018-19522-8 PMC5789839

[29] Weksler, B. B. et al. Blood-brain barrier-specific properties of a human adult brain endothelial cell line. *FASEB J.* **19**, 1872–1874 (2005). 16141364 10.1096/fj.04-3458fje

[30] Goyal, D. & Goyal, R. Angiogenic transformation in human brain micro endothelial cells: Whole genome DNA methylation and transcriptomic analysis. *Front. Physiol.* **10**, 1502 (2019). 31920707 10.3389/fphys.2019.01502 PMC6917667

[31] Auerbach, R., Lewis, R., Shinners, B., Kubai, L. & Akhtar, N. Angiogenesis assays: A critical overview. *Clin. Chem.* **49**, 32–40 (2003). 12507958 10.1373/49.1.32

[32] Zhang, J. et al. GJA1 gene polymorphisms and topographic distribution of cranial MRI lesions in cerebral small vessel disease. *Front. Neurol.* **11**, 583974 (2020). 33324328 10.3389/fneur.2020.583974 PMC7723976

[33] Krolak, T. et al. Brain endothelial gap junction coupling enables rapid vasodilation propagation during neurovascular coupling. *Cell* **188**, 5003-5019.e22 (2025). 40675149 10.1016/j.cell.2025.06.030 PMC12337775

[34] Sladojevic, N., Yu, B. & Liao, J. K. Regulator of G‐Protein Signaling 5 maintains brain endothelial cell function in focal cerebral ischemia. *J. Am. Heart Assoc.* **9**, e017533 (2020). 32875943 10.1161/JAHA.120.017533 PMC7726987

[35] Phillips, C. M., Johnson, A. M., Stamatovic, S. M., Keep, R. F. & Andjelkovic, A. V. 20 kDa isoform of connexin-43 augments spatial reorganization of the brain endothelial junctional complex and lesion leakage in cerebral cavernous malformation type-3. *Neurobiol. Dis.* **186**, 106277 (2023). 37652184 10.1016/j.nbd.2023.106277

[36] Kim, S. et al. Identification of a novel fusion iduronidase with improved activity in the cardiovascular system. *Mol. Genet. Metab. Rep.* **33**, 100917 (2022). 36159322 10.1016/j.ymgmr.2022.100917 PMC9489536

[37] Jia, G., Aroor, A. R., Jia, C. & Sowers, J. R. Endothelial cell senescence in aging-related vascular dysfunction. *Biochim. et Biophys. Acta (BBA)* **1865**, 1802–1809 (2019). 10.1016/j.bbadis.2018.08.008 31109450

[38] Palikuqi, B. et al. Adaptable haemodynamic endothelial cells for organogenesis and tumorigenesis. *Nature* **585**, 426–432 (2020). 32908310 10.1038/s41586-020-2712-z PMC7480005

[39] Workman, M. J. & Svendsen, C. N. Recent advances in human iPSC-derived models of the blood-brain barrier. *Fluids Barriers CNS* **17**, 30 (2020). 32321511 10.1186/s12987-020-00191-7 PMC7178976

[40] Vatine, G. D. et al. Modeling psychomotor retardation using iPSCs from MCT8-deficient patients indicates a prominent role for the blood-brain barrier. *Cell Stem Cell* **20**, 831-843.e5 (2017). 28526555 10.1016/j.stem.2017.04.002 PMC6659720

[41] Qian, T. et al. Directed differentiation of human pluripotent stem cells to blood-brain barrier endothelial cells. *Sci. Adv.* **3**, e1701679 (2017). 29134197 10.1126/sciadv.1701679 PMC5677350

[42] Linville, R. M. et al. Long-term cryopreservation preserves blood-brain barrier phenotype of iPSC-derived brain microvascular endothelial cells and three-dimensional microvessels. *Mol. Pharm.* **17**, 3425–3434 (2020). 32787285 10.1021/acs.molpharmaceut.0c00484 PMC9923881

[43] Lee, C. A. A. et al. Modeling and rescue of defective blood-brain barrier function of induced brain microvascular endothelial cells from childhood cerebral adrenoleukodystrophy patients. *Fluids Barriers CNS* **15**, 9 (2018). 29615068 10.1186/s12987-018-0094-5 PMC5883398

[44] CZI Cell Science Program et al. CZ CELLxGENE Discover: a single-cell data platform for scalable exploration, analysis and modeling of aggregated data. *Nucleic Acids Res* **53**, D886–D900 (2025). 39607691 10.1093/nar/gkae1142 PMC11701654

[45] Griffon, N., Jeanneteau, F., Prieur, F., Diaz, J. & Sokoloff, P. CLIC6, a member of the intracellular chloride channel family, interacts with dopamine D(2)-like receptors. *Brain. Res. Mol. Brain. Res.* **117**, 47–57 (2003). 14499480 10.1016/s0169-328x(03)00283-3

[46] Rieck, S. et al. Forward programming of human induced pluripotent stem cells via the ETS variant transcription factor 2: Rapid, reproducible, and cost-effective generation of highly enriched, functional endothelial cells. *Cardiovasc. Res.* **120**, 1472–1484 (2024). 38916487 10.1093/cvr/cvae129

[47] Zhang, H., Yamaguchi, T. & Kawabata, K. The maturation of iPS cell-derived brain microvascular endothelial cells by inducible-SOX18 expression. *Fluids. Barriers. CNS.* **20**, 10 (2023). 36732767 10.1186/s12987-023-00408-5 PMC9893670

[48] Grath, A. & Dai, G. SOX17/ETV2 improves the direct reprogramming of adult fibroblasts to endothelial cells. *Cell Rep. Methods* **4**, 100732 (2024). 38503291 10.1016/j.crmeth.2024.100732 PMC10985233

[49] Fetsko, A. R., Sebo, D. J. & Taylor, M. R. Brain endothelial cells acquire blood-brain barrier properties in the absence of Vegf-dependent CNS angiogenesis. *Dev. Biol.* **494**, 46–59 (2023). 36502932 10.1016/j.ydbio.2022.11.007 PMC9870987

[50] Wang, Q. et al. Activation of Wnt/β-catenin pathway mitigates blood-brain barrier dysfunction in Alzheimer’s disease. *Brain* **145**, 4474–4488 (2022). 35788280 10.1093/brain/awac236 PMC9762951

[51] Gumina, R. J., Kirschbaum, N. E., Piotrowski, K. & Newman, P. J. Characterization of the human platelet/endothelial cell adhesion molecule-1 promoter: Identification of a GATA-2 binding element required for optimal transcriptional activity. *Blood* **89**, 1260–1269 (1997). 9028949

[52] Yuan, L. et al. ETS-related gene (ERG) controls endothelial cell permeability via transcriptional regulation of the claudin 5 (CLDN5) gene. *J. Biol. Chem.* **287**, 6582–6591 (2012). 22235125 10.1074/jbc.M111.300236 PMC3307294

[53] Garry, D. J. Etv2 is a master regulator of hematoendothelial lineages. *Trans. Am. Clin. Climatol. Assoc.* **127**, 212–223 (2016). 28066054 PMC5216469

[54] Nuñez, J. K. et al. Genome-wide programmable transcriptional memory by CRISPR-based epigenome editing. *Cell* **184**, 2503-2519.e17 (2021). 33838111 10.1016/j.cell.2021.03.025 PMC8376083

[55] Li, Y. B. et al. Angiogenesis driven extracellular matrix remodeling of 3D bioprinted vascular networks. *Bioprinting* **30**, e00258 (2023).

[56] Zhao, S. et al. PiggyBac transposon vectors: The tools of the human gene encoding. *Transl. Lung Cancer Res.* **5**, 120–125 (2016). 26958506 10.3978/j.issn.2218-6751.2016.01.05 PMC4758974

[57] Galvan, D. L. et al. Genome-wide mapping of PiggyBac transposon integrations in primary human T cells. *J. Immunother.* **32**, 837–844 (2009). 19752750 10.1097/CJI.0b013e3181b2914c PMC2796288

[58] Love, M. I., Huber, W. & Anders, S. Moderated estimation of fold change and dispersion for RNA-seq data with DESeq2. *Genome Biol.* **15**, 550 (2014). 25516281 10.1186/s13059-014-0550-8 PMC4302049

[59] Pagès, H., Carlson, M., Falcon, S. & Li, N. AnnotationDbi: Manipulation of SQLite-based annotations in bioconductor. *R Packag.*10.18129/B9.bioc.AnnotationDbi (2025).

[60] Blighe, K., Rana, S. & Lewis, M. EnhancedVolcano: Publication-ready volcano plots with enhanced colouring and labeling. *R Packag.*10.18129/B9.bioc.EnhancedVolcano (2025).

[61] Yu, G., Wang, L.-G., Han, Y. & He, Q.-Y. ClusterProfiler: An R package for comparing biological themes among gene clusters. *OMICS* **16**, 284–287 (2012). 22455463 10.1089/omi.2011.0118 PMC3339379

[62] Yu, G. enrichplot: visualization of functional enrichment result. *R Packag.*10.18129/B9.bioc.enrichplot (2025).

