# Supplementary material for: Accelerated reprogramming of hiPSCs into functional brain endothelial-like cells using multiplexed CRISPR activation
Source: Sci Rep. 2026 Apr 9;16:16731. doi: 10.1038/s41598-026-46961-5 (PMC13223295; doi:10.1038/s41598-026-46961-5)
Supplement: Supplementary file 5 — Supplementary Information 5. [file 41598_2026_46961_MOESM5_ESM.pdf]

Supplementary Figure S1:

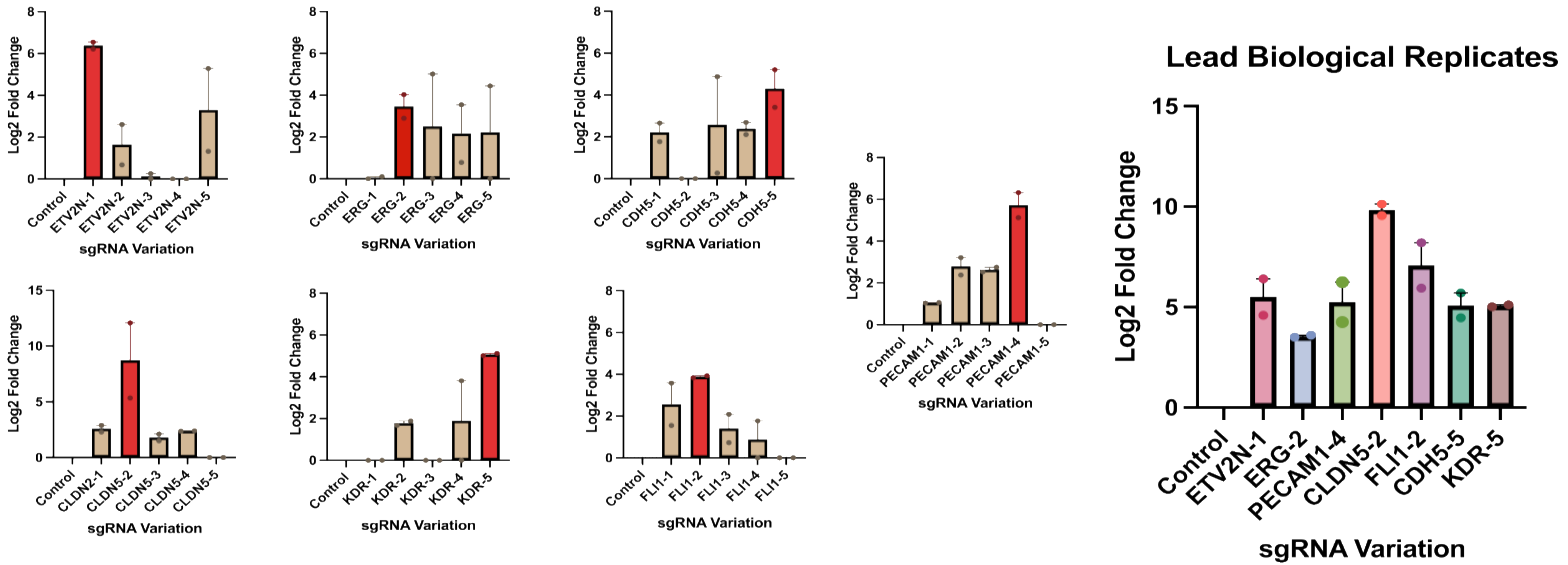

Supplementary Figure: S2

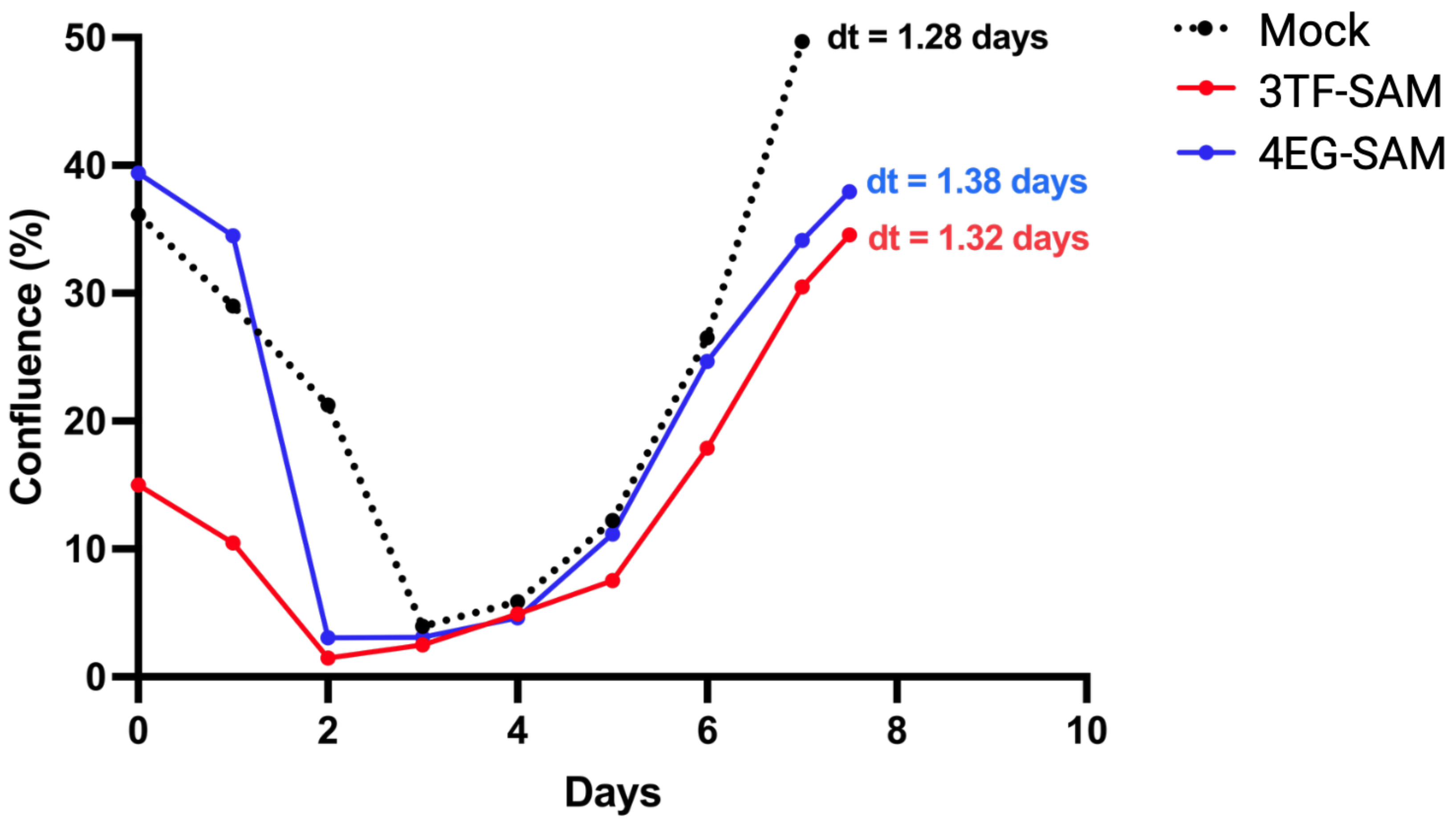

Supplementary Figure S3:

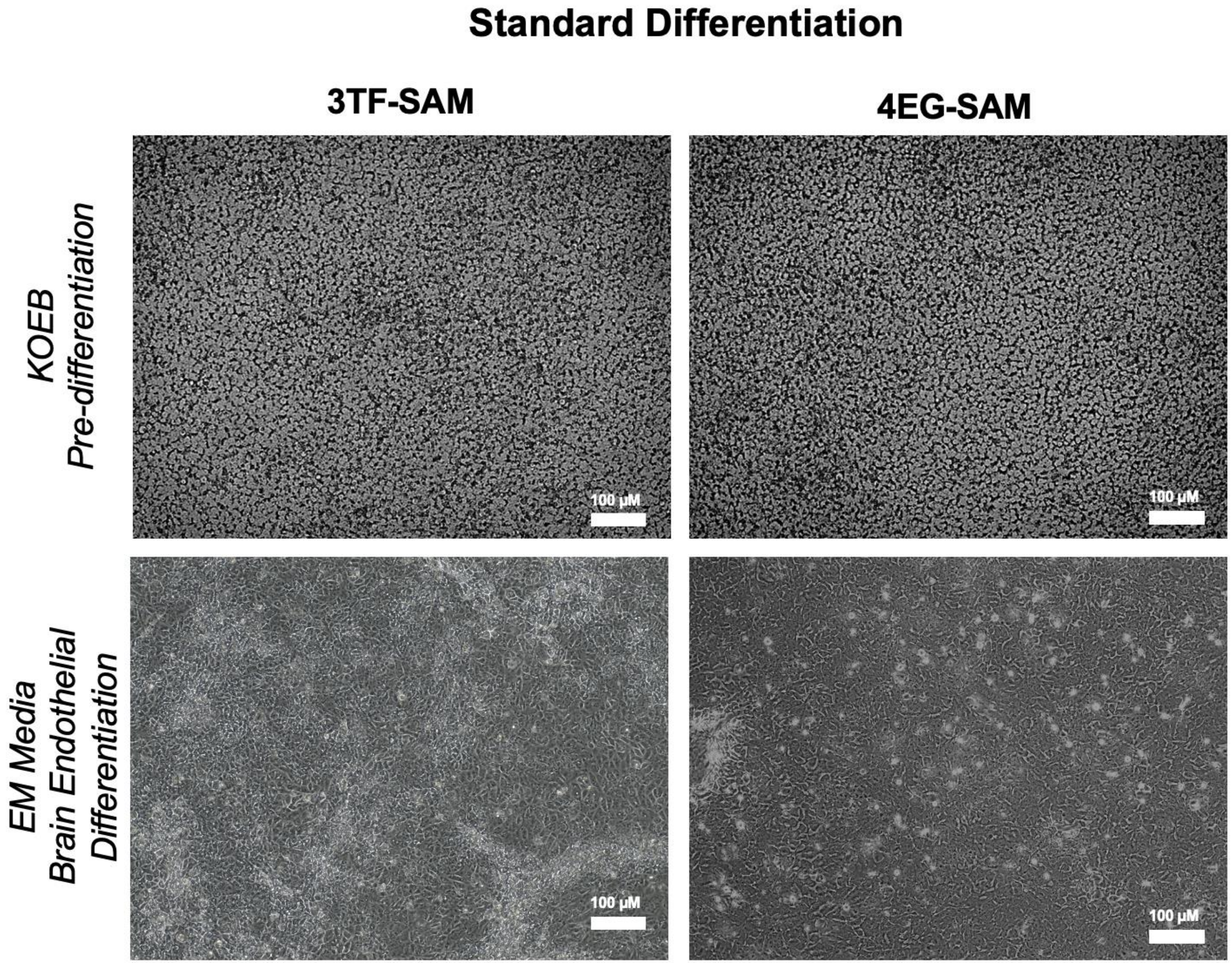

Supplementary Figure S4:

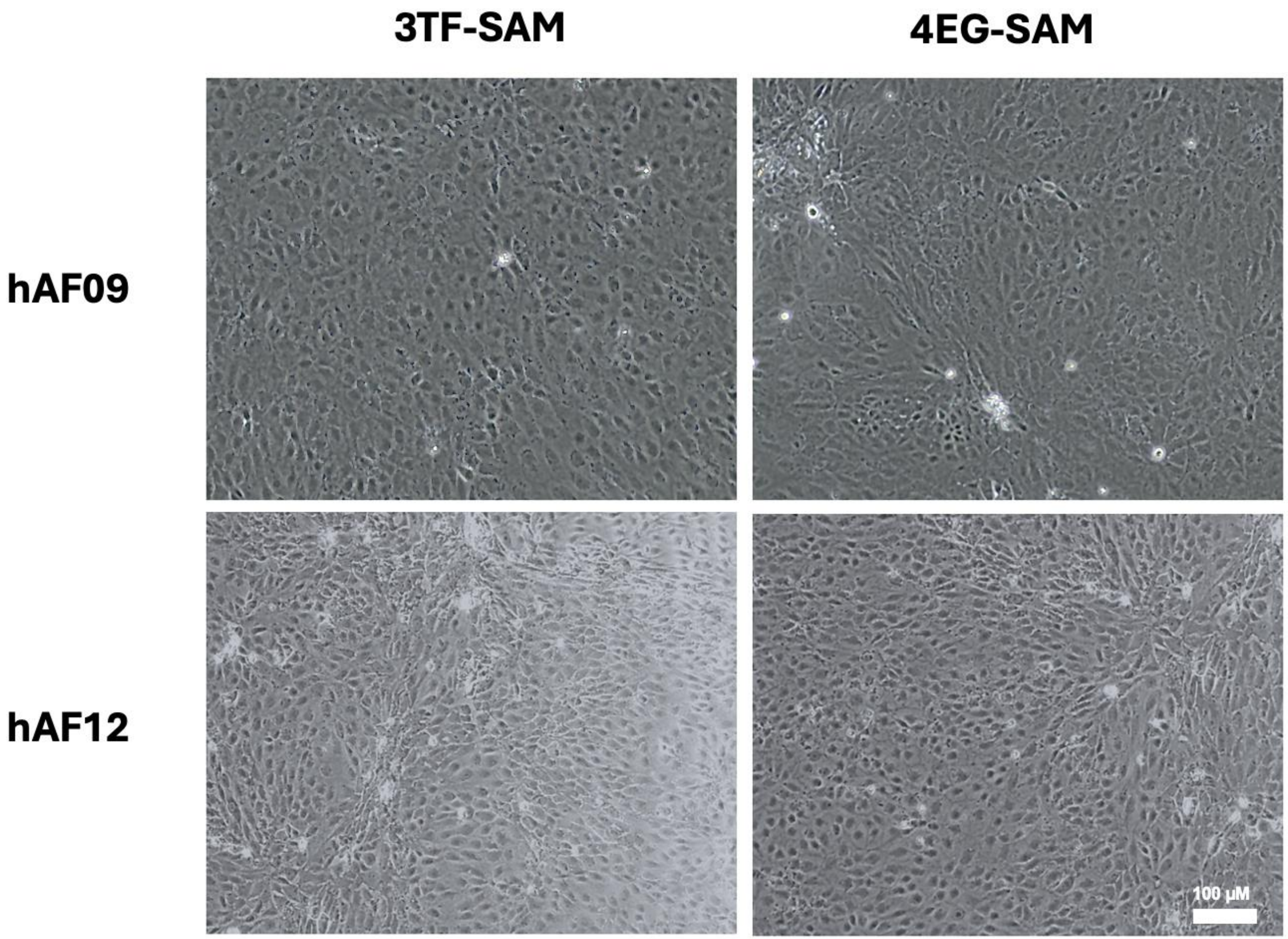

Supplementary Figure S5:

Uncropped dCas9 Expression in hAF

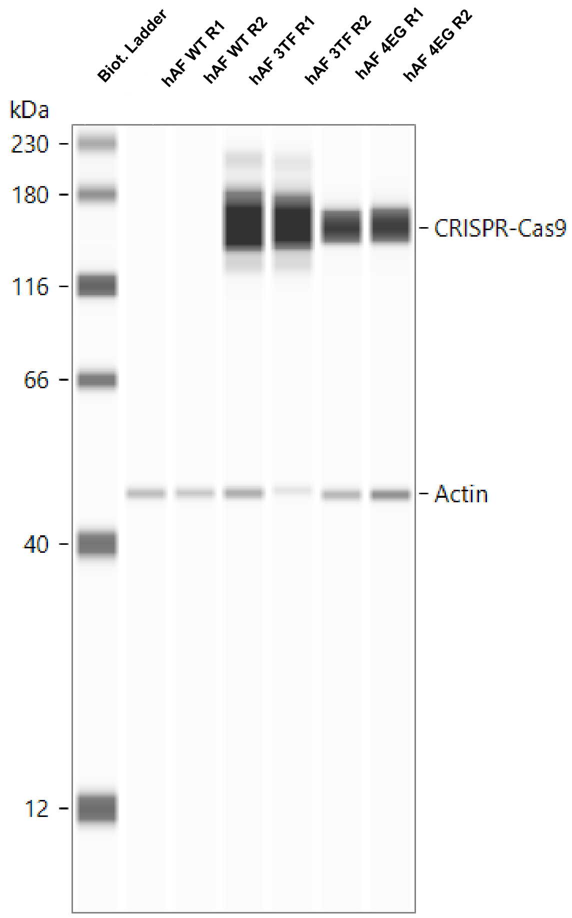

Uncropped dCas9 Expression in Various Parental hiPSCs

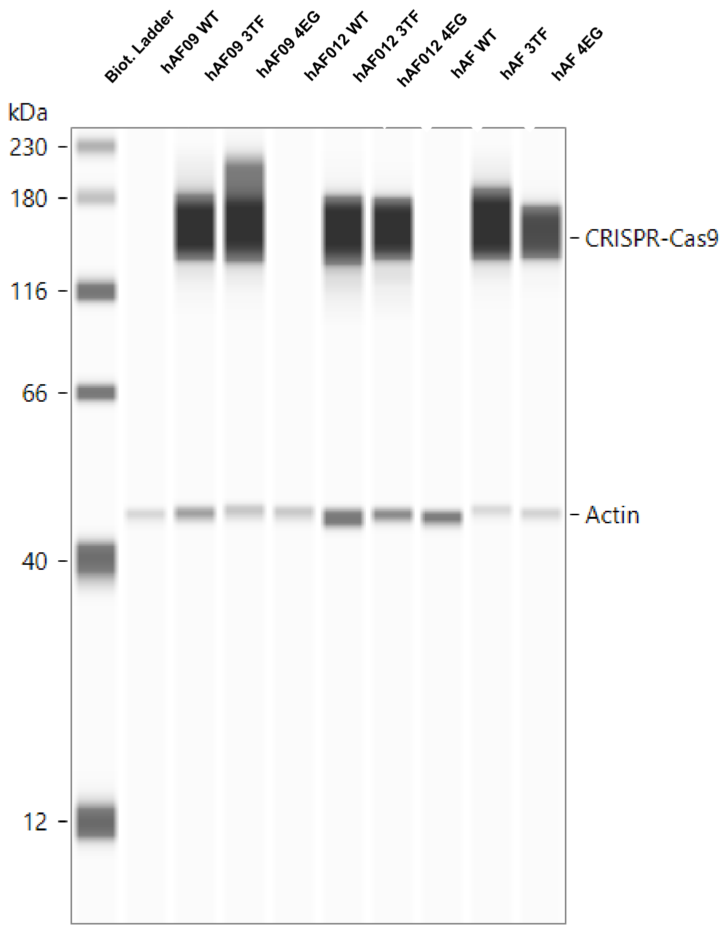

Supplementary Figure S6:

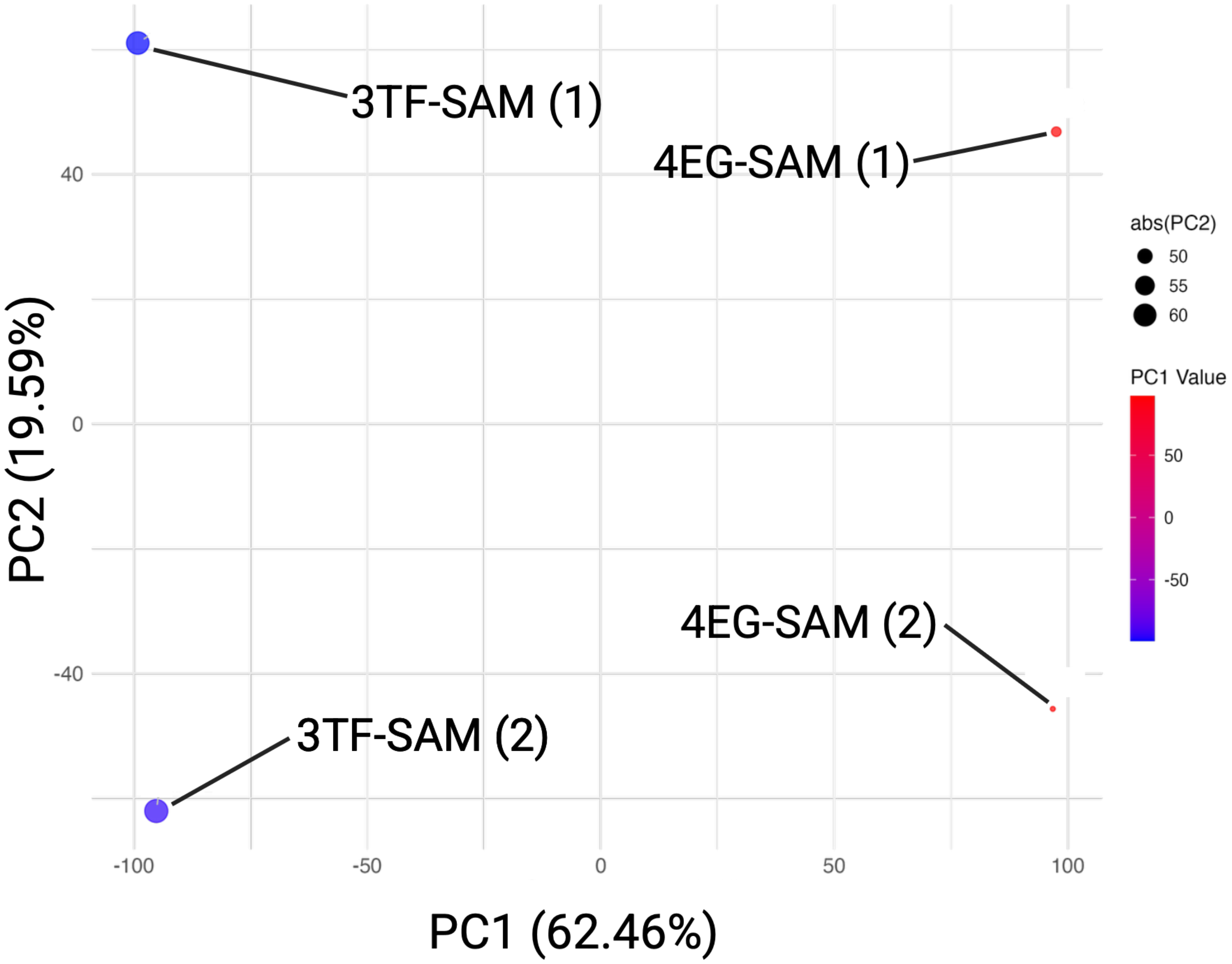

Supplementary Figure S7:

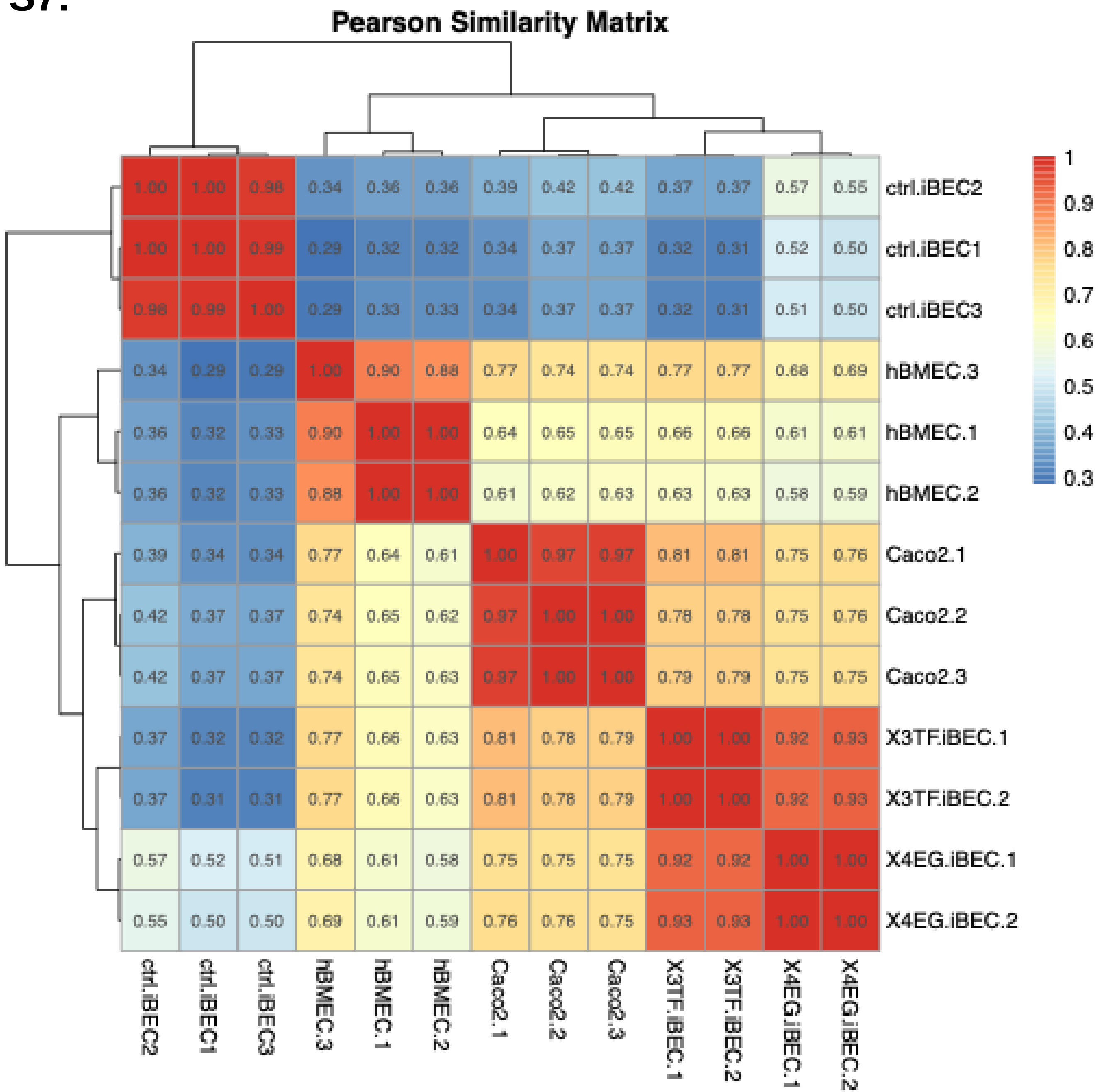

Supplementary Figure S8:

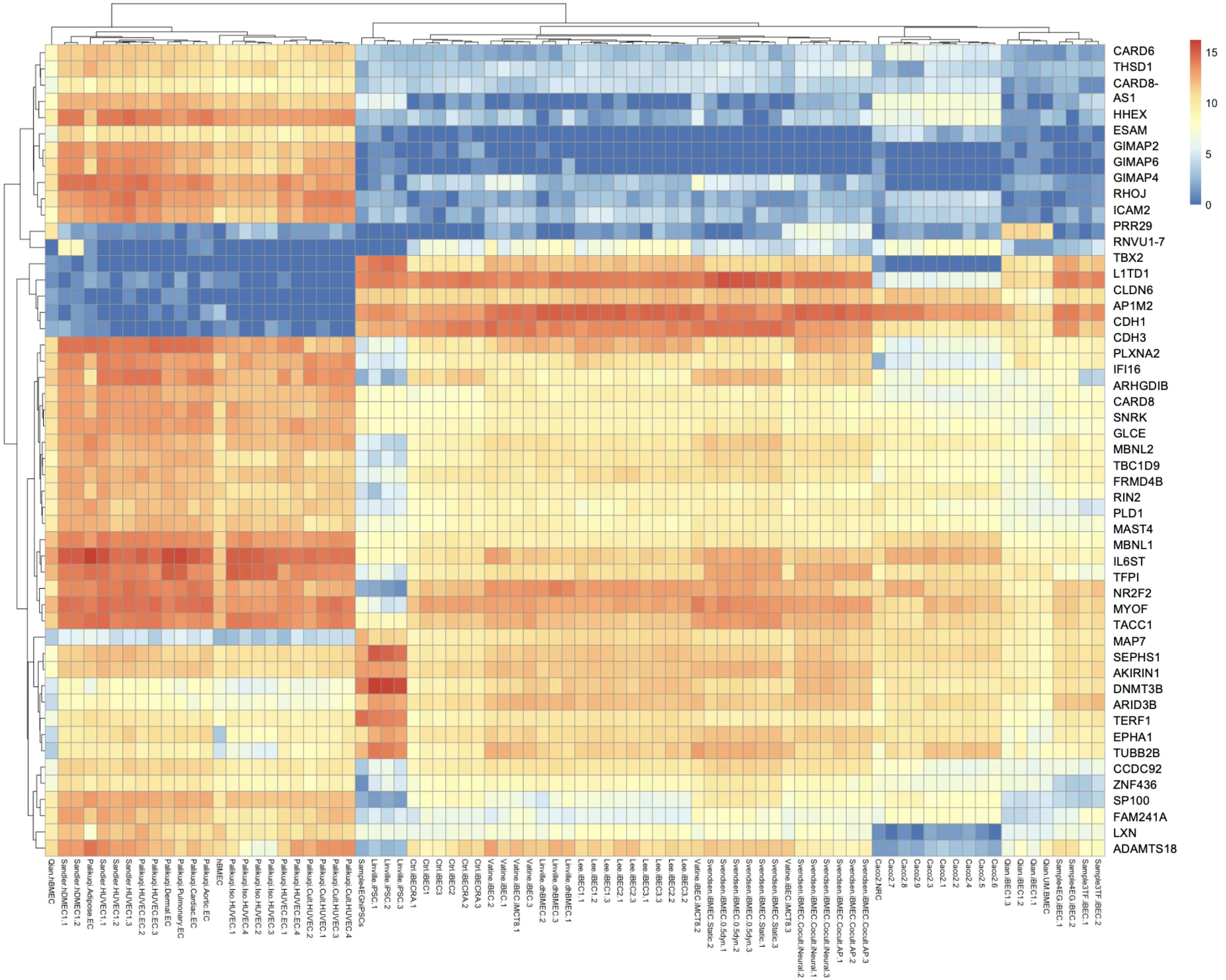

Supplementary Figure S9:

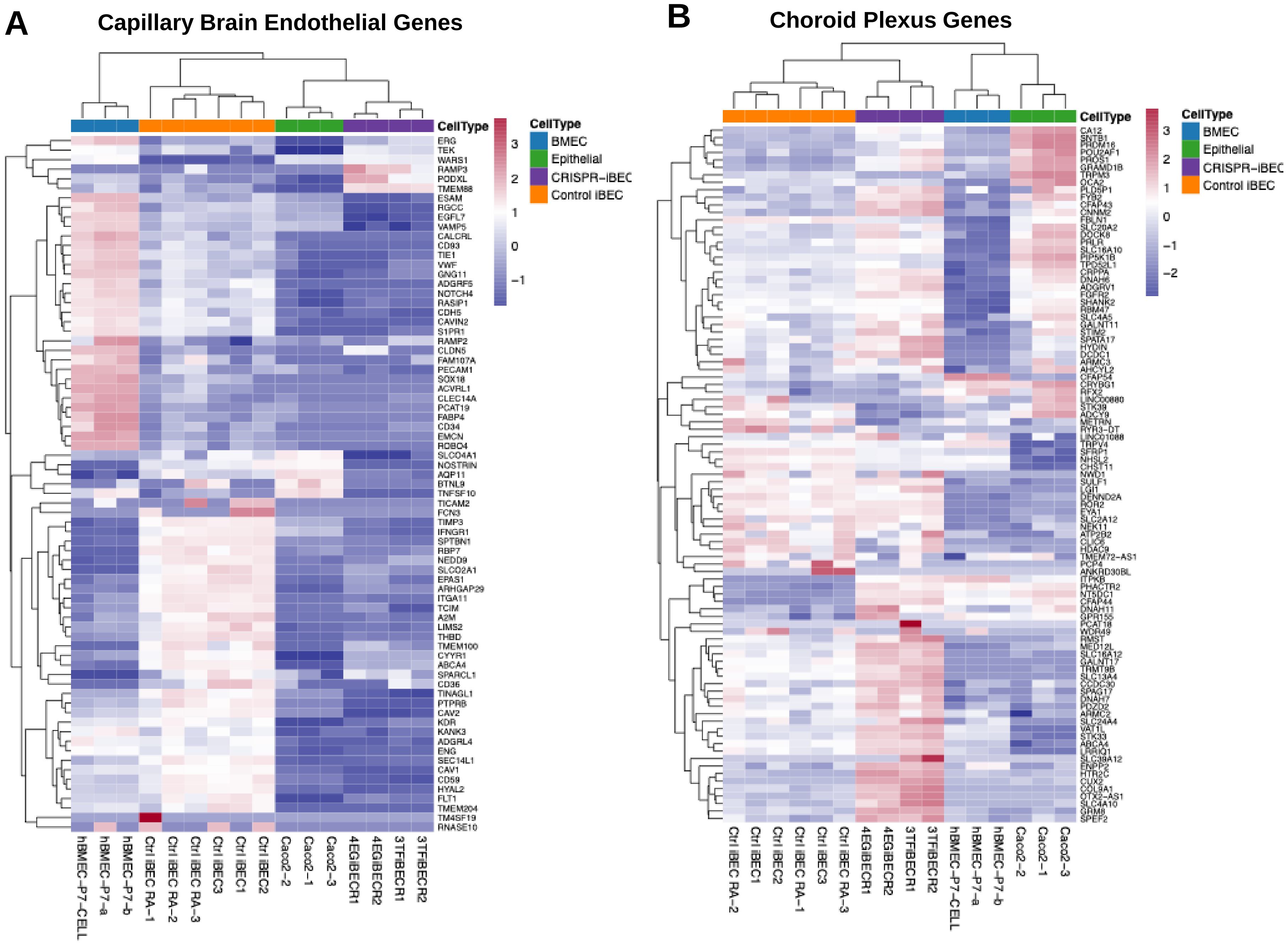

Supplementary Figure S10:

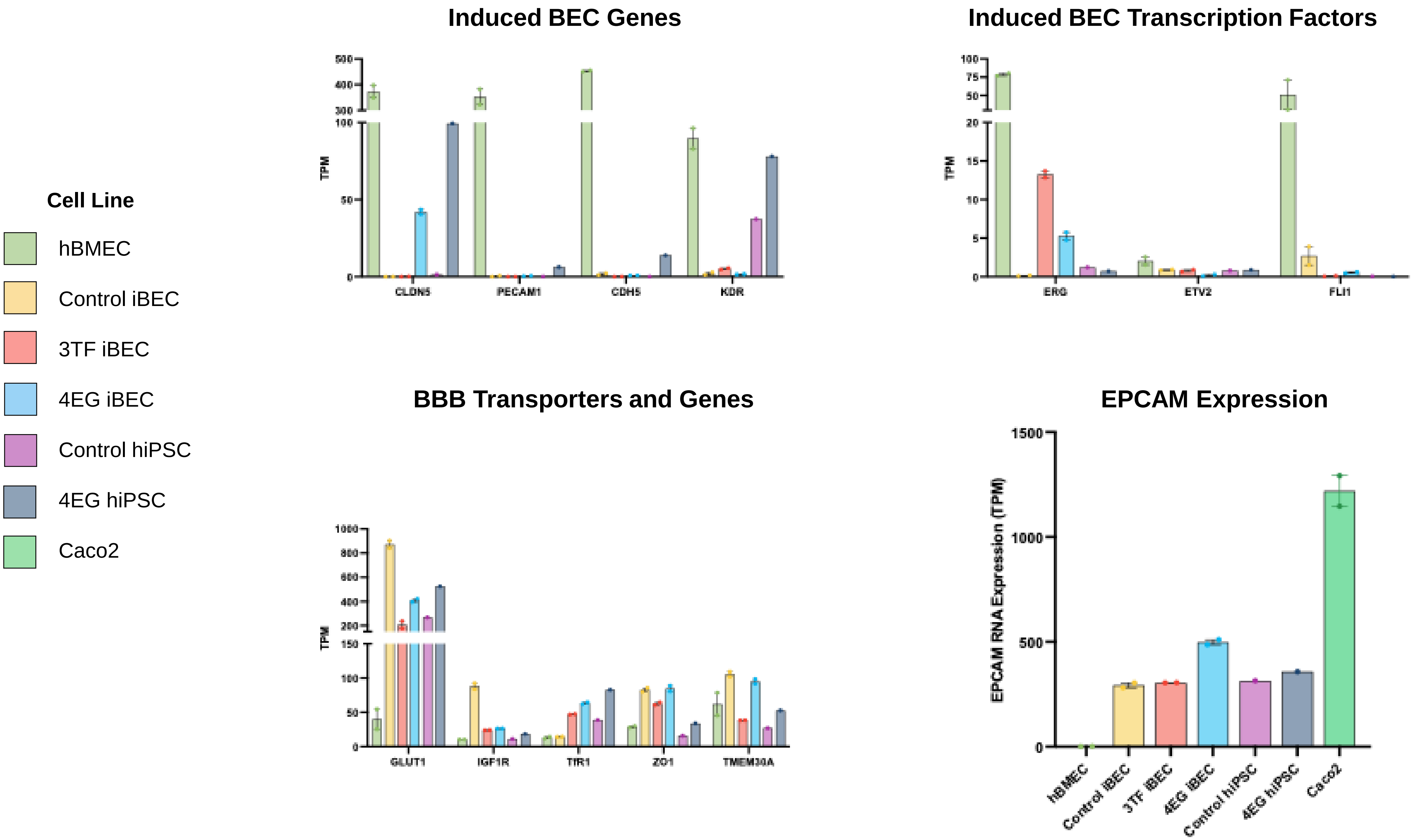

Table S1: Validated Highly Performing sgRNA Sequence Information

Gene Symbol	sgRNA Identity	Spacer Sequence (5′ to 3′)	mRNA ID
CLDN5	sgRNA1	GGCCGCGACTAGGACAACGG	NM_001130861.1
**CLDN5**	**sgRNA2**	**ACCCGCCGTTGTCCTAGTCG**	**NM_001130861.1**
CLDN5	sgRNA3	CCGCCCCCGAACCTTCAAAG	NM_001130861.1
CLDN5	sgRNA4	CCTTCAAAGAGGGTACCCCC	NM_001130861.1
CLDN5	sgRNA5	CTCGGCCGCGACTAGGACAA	NM_001130861.1
CDH5	sgRNA1	ATAACAGGAAACCATCCCAG	NM_001136154
CDH5	sgRNA2	GGCTCCCCTCCAAAGACGGT	NM_001136154
CDH5	sgRNA3	AGCCAGCCCAGCCCTCACAA	NM_001136154
CDH5	sgRNA4	GGCCAGCTGGAAAACCTGAA	NM_001136154
**CDH5**	**sgRNA5**	**TCTGTGGAGACAGCCATCCG**	**NM_001136154**
KDR	sgRNA1	GCCGAAACTCTAGAGCGCGG	NM_002253.4
KDR	sgRNA2	GGTGCCGAAACTCTAGAGCG	NM_002253.4
KDR	sgRNA3	CAGCGCAGTCCAGTTGTGTG	NM_002253.4
KDR	sgRNA4	CCAGCGCAGTCCAGTTGTGT	NM_002253.4
**KDR**	**sgRNA5**	**CCCCTCACCCGGGTACCCGG**	**NM_002253.4**
PECAM1	sgRNA1	AGGGAGGGTCAAGAACGCCA	NM_000442.5
PECAM1	sgRNA2	GCTGGAAACCGGGAACAATG	NM_000442.5
PECAM1	sgRNA3	GGAAGGCCGGTCATTTCCTG	NM_000442.5
**PECAM1**	**sgRNA4**	**AACAAAGCGGCCGCGTTCGG**	**NM_000442.5**
PECAM1	sgRNA5	GCAAAAGCCCTCTTTGATGG	NM_000442.5
FLI1	sgRNA1	TGACTTCACTTTGCGAATCG	NM_001167681.3
**FLI1**	**sgRNA2**	**CTTCACTTTGCGAATCGGGG**	**NM_001167681.3**
FLI1	sgRNA3	ACACACTATATAAATATACG	NM_001167681.3
FLI1	sgRNA4	TAGTGTGTGATGCGAAAAGC	NM_001167681.3
FLI1	sgRNA5	CACACAGGATACTTGCTCCT	NM_001167681.3
**ETV2**	**sgRNA1**	**ATTGTGACGTCAGCTGACGCTGG**	**NM_001300974.2**
ETV2	sgRNA2	CAATCGGCAGAGTAACTGCGGGG	NM_001300974.2
ETV2	sgRNA3	TTACAGCGCGGAAGGGCATGGGG	NM_001300974.2
ETV2	sgRNA4	CTAAACTATTACAGCGCGGAAGG	NM_001300974.2
ETV2	sgRNA5	ATTACAGCGCGGAAGGGCATGGG	NM_001300974.2
ERG	sgRNA1	GACTCCGCATGGCTTCACAC	NM_001136154.1
**ERG**	**sgRNA2**	**TGTGTCACACGGCGGCACAC**	**NM_001136154.1**
ERG	sgRNA3	CAGTGCAGTGTGTCACACGG	NM_001136154.1
ERG	sgRNA4	GACACACTGCACTGAGACCA	NM_001136154.1
ERG	sgRNA5	ACTGCCACAGCAGGTGTGAC	NM_001136154.1

***Highlighted green** indicates validated and selected sgRNA for CRISPRa multiplex system.

Supplementary Table S2: List of Human Induced Pluripotent Stem Cells for the Study

Parental hiPSC Cell Lines	Derived Source	Reprogramming Method
**hAF**	Amniotic fluid	Episomal Vectors
**hAF09**	Amniotic fluid	Sendai Virus Vector Reprogramming
**hAF12**	Amniotic fluid	Sendai Virus Vector Reprogramming

Table S3: Primers used for PCR and Sequencing in this Study.

Primer	Sequence 5′ to 3′	Purpose
qCLDN5-F	ATTTCGCTTCCCCTCCAAGA	qPCR
qCLDN5-R	CCCTGCCGATGGAGTAAAGA	qPCR
qCDH5-F	ACGCCTCTGTCATGTACCAA	qPCR
qCDH5-R	ACGATCTCATACCTGGCCTG	qPCR
qERG-F	CAGAGGGGTGAAGAAGGAGG	qPCR
qERG-R	TCCCCAAATGTCCTGAGTCC	qPCR
qETV2-F	ACCCCGTAAACTTCTCCCAG	qPCR
qETV2-R	TTTTCCGAGACGTCAGAGCA	qPCR
qFLI1-F	GACCGAGTCGTCCATGTACA	qPCR
qFLI1-R	CAGTATTGTGATGCGGCTCC	qPCR
qKDR-F	TGGGGAAAGCATCGAAGTCT	qPCR
qKDR-R	TTCCGGTTCCCATCCTTCAA	qPCR
qPECAM-F	TCCCCTAAGAATTGCTGCCA	qPCR
qPECAM-R	CTGGGGAGAGTTCTTGGTGT	qPCR
hU6-F	GAGGGCCTATTTCCCATGATT	Sequencing
M13-R	CAGGAAACAGCTATGAC	Sequencing
M13-F	GTAAAACGACGGCCAG	Sequencing
T7-R	TAATACGACTCACTATAGGG	Sequencing
TetON-F	TTGATATGCTGCCTGCTGACG	Sequencing
CmR-R	TGCTCATGGAAAACGGTGTAACAAG	Sequencing
TRE-F	AGCTCGTTTAGTGAACCGTCAG	Sequencing
MS2-R	GCGGAAGGGATAGGATTACC	Sequencing
MP-Ins-F	CAAGTTTGTACAAAAAAGCAGGCTTAGA	Sequencing
MP-Ins-R	GAGCTGCTTCGATGGATTTATCAA C	Sequencing

Table S4: Immunocytochemistry Antibodies and Reagents

Antibody	Supplier	Source	Catalog Code
Alexa Fluor 488 Goat-Anti-Rabbit igG	Invitrogen	Secondary	A11034
Alexa Fluor 488 Goat-Anti-Mouse igG	Invitrogen	Secondary	A28175
Anti-Human CD144 VE-Cadherin	Invitrogen	Primary	53-1449-42
CLDN5-488	Invitrogen	Primary	352588+A6
ERG	AbCam	Primary	ab133264
ETV2	AbCam	Primary	ab181847
FLI1	Invitrogen	Primary	MA1-196
Cas9	AbCam	Primary	EPR18991
VEGFR2-647	AbCam	Primary	ab307529
CD31-APC Flow Antibody	Thermofisher	Primary	17-0319-42
ZO-1	Invitrogen	Primary	40-2200
GLUT1-AF647	BD Pharmacology	Primary	566580
CD31	AbCam	Primary	Ab28364
Rb mAb GLUT1-488	AbCam	Primary	Ab195359
VE-Cadherin ICC	AbCam	Primary	ab33168
Occludin	Invitrogen	Primary	331588
IGF1R	Cell Signalling	Primary	3027S
TFR1	Invitrogen	Primary	13-6800
TMEM30A	Thermofisher	Primary	BS-16576R
GLUT1	Abcam	Primary	Ab195359

Table S5: Brain Endothelial Genes and Transcription Factor sgRNA Designs and Genomic Location

Gene	**Chromosomal Location by sgRNA Rank**<br>(Derived from NCBI GRch38)
**CLDN5**	(1) Chr22:19524629:q11.21 (Sense)<br>(2) Chr22:19524624:q11.21 (Antisense)<br>(3) Chr22:19524433:q11.21 (Sense)<br>(4) Chr22:19524422:q11.21 (Sense)<br>(5) Chr22:19524632:q11.21 (Sense)
**PECAM1**	(1) Chr17:64390990:q24.1 (Sense)<br>(2) Chr17:64390851:q24.1 (Antisense)<br>(3) Chr17:64390897:q24.1 (Sense)<br>(4) Chr17:64390927:q24.1 (Sense)<br>(5) Chr17:64391152:q24.1 (Sense)
**CDH5**	(1) Chr16:66366555:q21 (Sense)<br>(2) Chr16:66366646:q21 (Sense)<br>(3) Chr16:66366528:q21 (Sense)<br>(4) Chr16:66366590:q21 (Sense)<br>(5) Chr16:66366468:q21 (Sense)
**KDR**	(1) Chr4:55125613:q12 (Antisense)<br>(2) Chr4:55125610:q12 (Antisense)<br>(3) Chr4:55125761:q12 (Sense)<br>(4) Chr4:55125762:q12 (Sense)<br>(5) Chr4:55125695:q12 (Antisense)
**ETV2**	(1) Chr19:35641681:q13.12 (Sense)<br>(2) Chr19:35641662:q13.12 (Antisense)<br>(3) Chr19:35641353:q13.12 (Sense)<br>(4) Chr19:35641345:q13.12 (Sense)<br>(5) Chr19:35641352:q13.12 (Sense)
**ERG**	(1) Chr21: 38498761:q22.2 (Antisense)<br>(2) Chr21: 38498705:q22.2 (Sense)<br>(3) Chr21: 38498713:q22.2 (Sense)<br>(4) Chr21: 38498719:q22.2 (Antisense)<br>(5) Chr21: 38498683:q22.2 (Antisense)

Table S6: Top 20 Most Differentially Expressed Genes in 3TF-SAM iBECs. List of the top 20 genes ranked by absolute $\log_2$ fold change in 3TF-SAM iBECs compared to control and adjusted p-values.

Gene Name	log_2FC	padj
**CDC42**	-6.2756587	< 2.2e-16
**COL12A1**	-7.8907305	< 2.2e-16
**CTSV**	4.73131208	< 2.2e-16
**GJA1**	5.08771513	< 2.2e-16
**KRT7**	-7.163648	< 2.2e-16
LUM	-8.7848612	< 2.2e-16
**TGM2**	-10.060168	< 2.2e-16
**TIMP3**	-7.8506617	< 2.2e-16
**TFPI2**	-7.5173176	< 2.2e-16
**RGS5**	7.52374714	< 2.2e-16
**SYT7**	5.12779629	< 2.2e-16
CLSTN2	-5.197212	< 2.2e-16
**TINAGL1**	-7.6455125	< 2.2e-16
**TUBB**	-3.4483418	< 2.2e-16
**H19**	-8.69459	< 2.2e-16
**ARSI**	-6.7431472	< 2.2e-16
GABRP	-5.3016504	2.06E-285
TUBB6	-4.1503542	2.33E-284
FN1	-5.5417892	1.82E-278
ANXA1	-3.9589258	1.45E-272

Table S7: Top 20 Most Differentially Expressed Genes in 4EG-SAM iBECs. List of the top 20 genes ranked by absolute log_2 fold change in 4EG-SAM iBECs compared to control and adjusted p-values.

Gene Name	log_2FC	padj
**CDC42**	-6.0320837	< 2.2e-16
COL3A1	-9.4986773	< 2.2e-16
**COL12A1**	-6.5706148	< 2.2e-16
**CTSV**	5.46665066	< 2.2e-16
**GJA1**	4.13614621	< 2.2e-16
**KRT7**	-5.5302885	< 2.2e-16
NTRK2	-5.9692103	< 2.2e-16
**TGM2**	-9.4751907	< 2.2e-16
**TIMP3**	-5.9593921	< 2.2e-16
**TFPI2**	-6.2216628	< 2.2e-16
**RGS5**	7.95434299	< 2.2e-16
**TINAGL1**	-5.7180416	< 2.2e-16
**TUBB**	-3.4799914	< 2.2e-16
**H19**	-6.6855521	< 2.2e-16
**ARSI**	-6.3998317	8.69E-302
**SYT7**	4.97254238	1.32E-295
TGOLN2	-3.8991555	8.92E-242
CRABP2	-4.7765059	7.29E-229
STOM	3.958206	6.51E-228
CSRP1	-5.6394096	2.95E-221

Table S8: Data Compilation of all Described Studies for Meta-analysis

Author	GSE
Lee_2018	GSE108012
Linnville_2020	GSE151976
Palikuqi_2020	GSE131039
Qian_2017	GSE97575
Sandler_2014	GSE57662
Svendsen_2019	GSE129290
Vatine_2017	GSE97324
Adam_2022	GSE217504
